# Psychedelics in Multiple Sclerosis: Mechanisms, Challenges, and Prospects for Neuroimmune Modulation and Repair

**DOI:** 10.3390/cells14231872

**Published:** 2025-11-26

**Authors:** Ivan Anchesi, Maria Francesca Astorino, Ivana Raffaele, Deborah Stefania Donato, Serena Silvestro, Aurelio Minuti, Marco Calabrò, Michele Scuruchi, Giovanni Luca Cipriano

**Affiliations:** 1 IRCCS Centro Neurolesi “Bonino-Pulejo”, Via Provinciale Palermo, Contrada Casazza, 98124 Messina, Italyserena.silvestro@irccsme.it (S.S.); giovanniluca.cipriano@irccsme.it (G.L.C.); 2 Department of Biomedical and Dental Sciences and Morpho Functional Imaging, University of Messina, 98125 Messina, Italy; 3 Department of Clinical and Experimental Medicine, University of Messina, Via C. Valeria, 98125 Messina, Italy

**Keywords:** psychedelic compounds, multiple sclerosis, HTR2A receptors, neuroinflammation, neuroplasticity, glial cells, BDNF, therapeutic strategies

## Abstract

Multiple Sclerosis (MS) therapies effectively modulate peripheral immune responses but largely fail to promote neural repair within the central nervous system. This review evaluates whether psychedelic compounds (PSYs), via 5-HT2A activation, can fill a critical therapeutic gap: the need for agents that simultaneously suppress neuroinflammation and promote regeneration. We dissect the evidence suggesting PSYs can reprogram the neuroimmune milieu by downregulating key pro-inflammatory cytokines (e.g., TNF-α, IL-6) in glial cells while concurrently upregulating crucial neurotrophic factors (e.g., BDNF) that promote synaptic plasticity and oligodendrocyte support. However, we argue that the current evidence, largely derived from non-specific inflammation models, is insufficient to predict clinical efficacy in an autoimmune disease like MS. We critically analyze the significant translational barriers—from cardiovascular and psychiatric risks to profound legal and ethical challenges—that temper the immediate clinical promise. Finally, we propose a forward-looking perspective, suggesting that the true value of PSYs may lie not in their direct clinical use, but in uncovering novel therapeutic pathways. The emergence of non-hallucinogenic, functionally selective 5-HT2A agonists, inspired by psychedelic pharmacology, represents a more viable strategy to harness these mechanisms for MS therapy, demanding rigorous preclinical validation in disease-relevant models.

## 1. Introduction

Multiple Sclerosis (MS) is a chronic autoimmune demyelinating disease of the central nervous system (CNS), characterized by focal neuroinflammation, progressive axonal injury, and cumulative neurological deficits [1]. Standard disease-modifying therapies primarily aim to suppress immune responses, reduce relapse frequency and delay disease progression [2]. However, their efficacy is often partial, and many patients continue to accumulate lesions over time despite treatment [3]. Moreover, current therapies do not adequately address neural repair, underscoring the urgent need for novel strategies that combine immunomodulation with neuronal regeneration [4,5].

In recent years, a growing body of research has explored the potential of psychedelic compounds, both classic serotonergic psychedelics (PSYs) and non-hallucinogenic analogs, as therapeutic agents for neuroimmune disorders, including MS [6]. Classic serotonergic PSYs, such as lysergic acid diethylamide (LSD), psilocybin, and N,N-dimethyltryptamine (DMT), have gained attention in clinical neuroscience due to their ability to modulate brain plasticity, inflammation, and mood disorders [7,8,9]. Compounds like ketamine and 3,4-methylenedioxymethamphetamine (MDMA) have shown rapid and sustained benefits in treatment-resistant psychiatric conditions. Ketamine acts through glutamate receptor antagonism and downstream neuroplasticity mechanisms, whereas MDMA operates primarily via monoaminergic pathways [10,11].

PSYs exert their primary effects through serotonin 5-hydroxytryptamine (5-HT2A) receptors, which are expressed not only in cortical neurons but also in glial and immune cells [12,13,14]. Activation of this receptor, along with Sigma-1 (S1R) has been shown to reduce pro-inflammatory cytokines such as tumor necrosis factor-alpha (TNF-α) and interleukin-6 (IL-6), while enhancing anti-inflammatory responses, including the upregulation of IL-10. These actions shift the CNS environment toward a less inflammatory state, promoting remyelination [15,16,17]. Additionally, PSYs stimulate the release of neurotrophic factors like brain-derived neurotrophic factor (BDNF) and glial cell line-derived neurotrophic factor (GDNF) and activate intracellular signaling cascades such as mammalian target of rapamycin (mTOR) and Tropomyosin receptor kinase B (TrkB), which in turn promote synaptogenesis and cellular repair [18,19].

While much of the preclinical data originates from models of general inflammation or psychiatric conditions, these findings raise the question of whether such neurobiological effects could translate into structural changes in the CNS of MS patients. An isolated, yet intriguing, case study involving ibogaine anecdotally reported decreased lesion size and imaging biomarkers consistent with remyelination in an individual with MS [20]. Although this observation is limited by its anecdotal nature and the atypical, cardiotoxic profile of ibogaine, it serves to formulate the provocative hypothesis that psychedelic compounds may possess a regenerative potential in demyelinating disorders, warranting a more systematic investigation.

Despite these promising developments, several challenges must be addressed before PSYs can be integrated into clinical practice. Regulatory restrictions, safety concerns, and the need for supervised administration create significant barriers. However, the resurgence of scientific interest and the increasing number of Food and Drug Administration FDA-approved trials signal a shift in the paradigm of the perception and application of these compounds [21,22].

The rationale for investigating PSYs in MS lies in their dual mechanism: reducing neuroinflammation via immunomodulatory effects on microglia, astrocytes, and immune signaling, and promoting repair and plasticity via neurotrophic and synaptogenic pathways. This combination is particularly relevant in this disease, where inflammatory damage and neurodegeneration coexist, and where an ideal treatment should aim to both suppression of immune attacks on myelin and CNS regeneration [15,17]. This narrative review synthesizes current evidence on how PSYs may modulate the neuroimmune environment in MS, enhance neuroregenerative processes, and overcome existing therapeutic limitations. Particular attention will be given to the genes and the related biological pathways underlying the action of PSYs. Finally, by reviewing both preclinical and clinical findings, we will highlight the translational challenges and future prospects for integrating PSYs into MS therapy.

## 2. Comparative Summary of Psychedelic Classes and Rationale

Classical PSYs such as LSD and psilocybin primarily act via HTR2A activation [23], inducing cortical glutamate release and AMPA-mediated neurotransmission, which enhances neuroplasticity and synaptogenesis [12,16]. These mechanisms also modulate neuroimmune responses by reducing the release of pro-inflammatory cytokines (such as TNF-α and IL-1β) from immune cells, and by influencing the activity of astrocytes and microglia, both of which are implicated in MS-related neuroinflammation [15,16,24,25]. Psilocybin effect on synaptic vesicle protein 2A (SV2A) suggests sustained synaptic remodeling [26]. However, 5-HT2B receptor affinity in some PSYs (e.g., LSD) poses cardiotoxic risks, complicating their use [27].

Ketamine, an NMDA receptor antagonist, reduces excitotoxicity which acts as a driver of neurodegeneration in MS by limiting calcium influx and modulating glutamatergic signaling. It alters the kynurenine pathway to mitigate inflammation-linked neurotoxicity, a mechanism demonstrated by its ability to prevent the reduction of hippocampal neurogenesis induced by pro-inflammatory cytokines (such as IL-1β) [28] and can reset the HPA axis, reducing stress-related neuronal damage. Ketamine’s psychoplastogenic effects promote neural plasticity and may indirectly support remyelination and cellular repair, although direct evidence in MS remains limited. It is interesting to note that while ketamine shows clear immunomodulatory effects [28,29,30,31], these appear to be selective. For instance, in patients undergoing cancer surgery, ketamine did not alter the cytotoxicity of Natural Killer (NK) cells, suggesting it does not globally suppress all immune responses, a potentially advantageous feature in the context of an autoimmune disease [32]. Its dual impact on excitotoxicity and inflammation highlights its unique positioning apart from serotonergic PSYs [28,33,34,35,36,37].

Emerging PSYs such as DMT, a serotonin-acting compound, and ibogaine, an atypical multi-receptor modulator, show distinct pharmacodynamic profiles. DMT functions chiefly as a 5-HT1A/2A/2C agonist (with isolated early reports of partial 5-HT2 antagonism), and MAO-A inhibition in ayahuasca markedly prolongs its CNS effects [11,38,39,40]. Ibogaine interacts with opioid and NMDA receptors, modulating neurotransmission and immune pathways. It affects astrocytic and microglial function and promotes plasticity, potentially creating a reparative environment in MS lesions [20]. All these compounds share the capacity to modulate glial responses, reduce inflammation, and enhance neuroplasticity, which represent promising, albeit clinically unproven, therapeutic avenues for potentially mitigating MS progression (Table 1). However, their clinical application is hindered by safety concerns (e.g., ibogaine cardiotoxicity, psychedelic-induced psychiatric effects), legal restrictions, and limited mechanistic clarity [15,38].

Synthetic analogs such as (R)-2,5-Dimethoxy-4-iodoamphetamine (DOI) elicit psychoplastogenic plasticity, yet data confirm hallucinogenic effects persist [34]. The integration of PSYs with existing MS therapies may enhance remyelination and reduce neuroinflammatory damage. Continued pharmacological refinement and multidisciplinary research are essential to validate and translate these compounds into effective MS treatments.

## 3. Search Strategy and Selection Criteria

This narrative review synthesizes and critically evaluates peer-reviewed literature concerning the intersection of psychedelic compounds and MS. A comprehensive literature search was conducted using the PubMed/MEDLINE database, supplemented by targeted searches on Google Scholar and AI-driven academic search engines to ensure broad coverage of this interdisciplinary field. The search focused on articles published up to June 2025, using keywords such as “psychedelics,” “psilocybin,” “LSD,” “DMT,” “multiple sclerosis,” “neuroinflammation,” “neuroplasticity,” “remyelination,” and “glial cells.” We prioritized original research articles (both preclinical and clinical), systematic reviews, and meta-analyses that provided mechanistic insights or translational data. The selection was guided by relevance to the core topics of neuroimmune modulation and neural repair in the context of MS. Commentaries, case reports (unless highly illustrative), and gray literature were generally excluded. The search was restricted to articles published in English.

## 4. Mechanisms of Psychedelic-Induced Neuroimmune Modulation

### 4.1. Inflammation and Cytokine Regulation

#### 4.1.1. Evidence from Animal Models

The primary targets of classical PSYs are serotonin (5-HT) receptors, particularly the 5-HT2A receptor, which plays a crucial role in both the psychoactive and immunological effects of these compounds [69,70]. Activation of 5-HT2A receptors significantly modulate a cascade of intracellular signals that can deeply alter immune functions [71,72]. One well-documented consequence is the modulation of cytokine production, which plays a pivotal role [71,72,73]. Psychedelic activation of the 5-HT2A receptor has been hypothesized to reduce pro-inflammatory cytokines such as TNF-α, which are key drivers of inflammation. Indeed, multiple studies now support this, showing that PSYs can suppress a wide array of pro-inflammatory mediators. As a proof-of-concept for their general anti-inflammatory properties, several studies have utilized models of systemic inflammation induced by lipopolysaccharide (LPS). In these models, which primarily test innate immune responses, compounds like Ayahuasca and DMT (in a rat model of LPS-induced depression) have been shown to significantly reduce levels of TNF-α, IL-1α and IL-12p70 [74]. In particular, psilocybin (in a systemic LPS-induced inflammation model) has demonstrated potent effects in downregulating LPS-induced IL-1β, IL-6, and Cyclooxygenase-2 (COX-2) mRNA levels in mouse liver [50], and other PSYs like MDMA have similarly been shown to attenuate stress-induced IL-1β in the hippocampus of rats [59]. Psilocybin has also been found to regulate the IL-17a pathway, recently identified target in the modulation of immune responses. This connection is further supported by evidence in male rats that selective inhibition of IL-17a in the prefrontal cortex reduce heroin relapse in a ’heroin seeking’ model, suggesting a causal link between this inflammatory pathway and the behavioral effects of psilocybin [75].

#### 4.1.2. Evidence from Human Studies

Crucially, the effects of psilocybin have been further corroborated by human data. A placebo-controlled study in healthy volunteers demonstrated that psilocybin acutely reduced TNF-α concentrations and persistently lowered IL-6 and C-reactive protein (CRP) levels for up to seven days [60]. However, evidence in humans is not entirely consistent. Another study in healthy individuals did not observe statistically significant changes in hsCRP or TNF one day after a psilocybin dose. Although a non-significant 32% decrease in hsCRP was reported, TNF showed a numerical increase. The authors noted that the study may have been underpowered to detect small changes and that a single post-intervention blood sample might have missed the temporal dynamics of these biomarkers. This suggests that the anti-inflammatory effects of PSYs may be more pronounced in clinical cohorts with pre-existing inflammation [76]. Specific 5-HT2A receptor agonists have been hypothesized to exert anti-inflammatory effects, potentially through the inhibition of this signaling pathway [77]. Concurrently, there may be an upregulation of anti-inflammatory factors, such as IL-10, as observed, albeit non-significantly, in human macrophages treated with magic mushroom extracts [58], or a shift toward an anti-inflammatory cytokine profile. While these findings establish the potential for PSYs to modulate inflammatory pathways, it is crucial to note that these models do not reflect the complex, T-cell-driven autoimmune pathology of MS. Therefore, whether these immunomodulatory effects translate to a neuroprotective phenotype in the context of MS remains a critical open question that can only be addressed in disease-relevant preclinical models, such as Experimental Autoimmune Encephalomyelitis (EAE). However, it is crucial to note that these effects can be context- and dose-dependent. For instance, a recent study found that an extract of *Banisteriopsis caapi*, at the concentration used, induced a pro-inflammatory environment and promoted noradrenergic neuron depletion in a rodent model, highlighting that the outcome is not universally beneficial [78]. These discrepancies in human studies are likely attributable to methodological differences, including dosing, the timing of blood sampling (which may have missed key temporal dynamics), and the low baseline inflammatory state of healthy volunteers, which may mask an anti-inflammatory effect that would be more prominent in a clinical population with active inflammation.

#### 4.1.3. Receptor-Specific Mechanisms

5-HT2A receptor signaling in immune cells triggered by PSYs also influences the nuclear factor kappa B (NF-κB) pathway, which is recognized as a central hub in the regulation of inflammatory gene expression [46]. However, the universality of this mechanism remains debated, as a study on primary human T-lymphocytes and monocytes found that classic PSYs did not directly modulate NF-κB activation upon stimulation, suggesting that the effects may be cell-type specific or mediated through indirect mechanisms [79]. In contrast, other studies have reported that NF-κB may act as a downstream mediator of psilocin’s effects in microglia, as this transcription factor is known to regulate the expression of both NADPH oxidase (NOX) and inducible nitric oxide synthase (iNOS), which are responsible for ROS and NO production, respectively [15,46].

Evidence shows that certain 5-HT2A agonists with psychedelic properties, such as (R)-DOI (in primary rat aortic smooth muscle cells stimulated with TNF-α), can inhibit NF-κB nuclear translocation and suppress TNF-α-induced inflammatory gene expression in vitro, including ICAM-1, VCAM-1, and IL-6. These findings suggest that such compounds may exert potent anti-inflammatory effects by modulating immune-related signaling pathways [41]. Similarly, although ketamine primarily targets the glutaminergic system, it also exerts potent anti-inflammatory effects, underscoring immunomodulation as a critical component of its therapeutic action. In animal models of post-traumatic stress disorder (PTSD), S-ketamine has been shown to attenuate pro-inflammatory responses by reducing TNF-α and IL-1β levels in critical brain regions such as the striatum and periaqueductal gray [29]. Furthermore, R-ketamine has also been shown to attenuate delirium and cognitive impairment induced by high doses of LPS, reducing the release of inflammatory cytokines both systemically and centrally [80]. Another key mechanism likely involves the inhibition of the NLRP3 inflammasome; ketamine prevents depressive-like behaviors induced by inflammatory stressors potentially through the suppression of this signaling pathway [30]. These findings are consistent with earlier studies on selective 5-HT2A agonists such as (R)-DOI (2,5-dimethoxy-4-iodoamphetamine) [6,45]. In animal models of inflammation, (R)-DOI strongly attenuates inflammatory responses by blocking TNF-α-mediated inflammation in vivo and preventing downstream effects [42,81]. Adding another layer of complexity, recent studies highlight the concept of functional selectivity, whereby structurally similar 5-HT2A agonists can elicit different immunological outcomes. For example, (R)-DOI prevents inflammation in a rat asthma model, whereas the related compound (R)-DOTFM does not, despite comparable affinity for 5-HT2A receptors. This research identified suppression of arginase 1 (Arg1) expression as a key anti-inflammatory mechanism, independent of canonical signaling pathways [44]. Specifically, pretreatment with (R)-DOI blocked OVA-induced Arg1 expression, while (R)-DOTFM significantly increased Arg1 expression above that of the OVA-alone group, providing strong evidence for this differential mechanism [44].

Notably, these anti-inflammatory effects in rodents were observed at doses lower than those required to elicit overt behavioral changes, suggesting that 5-HT2A-mediated immunomodulation may occur independently of a full psychedelic experience.

Interestingly, a promising therapeutic mechanism of PSYs in MS involves the modulation of microglial activation states. Microglia, the resident immune cells of CNS, play a central role in sustaining chronic neuroinflammation and promoting tissue injury. These cells release pro-inflammatory cytokines such as TNF-α, IL-1β, and NO, thereby exacerbating demyelination and axonal degeneration [46,82,83]. This inflammatory cascade is further amplified by astrocyte-microglia interactions, contributing to neuronal damage [2,84].

PSYs compounds, such as psilocybin and its metabolite psilocin, have demonstrated the ability to shift microglia state from a pro-inflammatory to a more regulated or reparative phenotype [46]. Specifically, in vitro studies show that psilocybin and psilocin suppress TNF-α expression in LPS-activated microglia [85]. Beyond cytokines modulation, psilocin significantly inhibits key microglial effector functions, including phagocytic activity and the production of ROS and NO, in a 5-HT2R-dependent manner [46]. The context appears critical, as one study reported that psilocin reduced TNF-α levels in activated macrophages, on the contrary, both psilocybin and psilocin, could trigger its release in resting (unstimulated) cells, highlighting a nuanced, state-dependent activity [77,86]. It is also important to note that some studies used extracts from whole psilocybin-containing mushrooms, which, in addition to psilocybin, contain other mycochemicals like alkaloids and flavonoids that in turn may contribute to the psilocybin anti-inflammatory effects [56,58]. This action is reminiscent of dimethyl fumarate (DMF), an approved MS therapy, which also downregulates pro-inflammatory cytokines like IL-1β and TNF-α through microglial modulation [87,88].

Overall, PSYs may modulate the neuroimmune system through the activation of the 5-HT2A receptor, influencing cytokine production, microglial activity, and the NF-κB pathway. These actions contribute to reduce inflammation and promoting a more auspicious environment for neuronal repair, highlighting their potential therapeutic applications in MS. However, further research is needed to confirm these effects.

### 4.2. Neurotrophic Factors, Plasticity, and Cellular Repair

Beyond their immunomodulatory properties, PSYs have attracted attention for their capacity to stimulate neuroplasticity and neuronal regeneration [89].

Considering that MS involves significant neuronal damage and axonal loss, especially in advanced stages, therapeutic strategies aimed at neuronal repair and preservation of neural circuits are of considerable interest [90]. PSYs may support neuronal repair processes primarily by promoting neuron survival, axonal sprouting, dendritic branching, and synaptogenesis. These functions are mediated through the upregulation of neurotrophic factors and activation of intracellular growth signaling pathways.

In this setting, one of the most consistently observed effects of classic PSYs, such as LSD and DMT, is their ability to increase the expression of BDNF, a key molecule involved in neuronal survival and synaptic plasticity [8]. A superimposable effect has been observed for ketamine, which, despite being an NMDA receptor antagonist, converges on the same neurotrophic pathways. Furthermore, recent studies have confirmed that the synaptogenic effects of psilocin are comparable to those of ketamine, which specifically increases clusters of the post-synaptic protein PSD-95 [57]. Several studies have underlined the importance of the BDNF/TrkB pathway for ketamine’s action. By modulating this pathway ketamine (in a mouse model of post-stroke depression) can restore dendritic spine density and the expression of synaptic proteins such as PSD-95 and SYP during stroke induced neurological damage [31]. It is interesting to note that, in addition to BDNF, other neurotrophic factors such as IGF-1 (Insulin-like growth factor 1) are also implicated, acting as independent but essential mediators for the sustained effects of ketamine [47]. However, new research suggests this relationship is complex; for instance, in animal models of stress, psilocybin did not reverse the stress-induced decrease in BDNF [91]. In microglial models, psilocybin and psilocin have been shown to suppress pro-inflammatory cytokines and increase BDNF levels through 5-HT2A, 5-HT2B, 5-HT7, and TrkB signaling [85]. Their pro-plasticity effects are primarily mediated via 5-HT2A and TrkB signaling pathways. While the canonical view holds that these pro-plasticity effects are downstream of 5-HT2A activation, paradigm-shifting (though still debated) recent findings propose a more direct mechanism. This research suggests that some PSYs, such as LSD and psilocin, can directly bind to the TrkB receptor with high affinity, effectively mimicking BDNF [25,92]. In a striking departure from established models, these studies reported that the pro-plasticity effects were dependent on TrkB but independent of 5-HT2A activation [25]. If independently replicated, this finding would represent a fundamental revision of psychedelic pharmacology. However, the exact interplay between 5-HT2A-dependent and TrkB-direct mechanisms remains an area of intense investigation and is crucial for understanding how to pharmacologically separate therapeutic plasticity from hallucinogenic effects. Other research highlights that the plasticity-promoting properties are mediated by the activation of intracellular 5-HT2A receptors, which explains why serotonin itself, being less membrane-permeable, does not trigger similar plastic changes [51]. Blocking 5-HT2A with ketanserin or TrkB with ANA-12 completely abolishes psychedelic-induced neuritogenesis (dendritic growth) and spinogenesis. These findings suggest that certain PSYs may directly recruit TrkB, mimicking BDNF activity to stimulate dendritic and synaptic formation both in vitro and in vivo [49,89]. Furthermore, PSYs such as ibogaine robustly induce glial-derived neurotrophic factor (GDNF) expression, originally explored in addiction research but increasingly considered relevant for broader neuronal regenerative applications [19,20].

These neurotrophic effects correlate with structural neuronal changes observed in animal models, where PSYs have induced increases in dendritic spine density and complexity, including the upregulation of specific synaptic proteins like PSD95 and GAP43 [93], even after a single dose, a phenomenon termed “psychoplastogenic” effects [48,51]. Such structural remodeling supports neuronal resilience and enhances neural circuit flexibility.

Additionally, the mechanistic target of mTOR pathway, activated by PSYs via HTR2A receptor stimulation, is central to these regenerative processes, a mechanism confirmed by proteomic analyses in human cerebral organoids exposed to LSD [94,95]. The mTOR pathway regulates protein synthesis, cytoskeletal remodeling, and synapse formation, contributing to neuronal repair and rapid antidepressant effects observed with PSYs [51,96,97]. Activation of the mTOR pathway, together with PI3K/Akt signaling, fosters structural plasticity that counteracts neuronal atrophy and facilitates tissue repair [89].

At a network level, this induction of plasticity is thought to operate by reopening a “juvenile-like” critical period. As proposed by Brunello et al., the activation of TrkB receptors, particularly on parvalbumin-positive (PV+) interneurons, reduces cortical inhibition. This disinhibition of pyramidal neurons makes the entire network more malleable and receptive to being reshaped by environmental and therapeutic inputs, facilitating the rewiring of dysfunctional circuits [92].

### 4.3. Remyelination and Oligodendrocyte Support

Remyelination is a crucial yet often incomplete reparative process in MS, essential for restoring the myelin sheath around demyelinated axons [98]. Spontaneous remyelination by oligodendrocyte progenitor cells frequently fails or is insufficient, leaving chronic demyelinated lesions in many patients [99]. Therapeutic strategies enhancing remyelination by supporting oligodendrocyte function and differentiation remain critical unmet needs.

Emerging, albeit indirect, evidence suggests a hypothesis that PSYs might support remyelination by promoting oligodendrocyte progenitor cell differentiation and supporting myelin-forming oligodendrocytes. A preclinical study highlights a direct cellular impact, finding that a single administration of psilocybin induces the expression of the activity marker Fos in oligodendrocytes in the rat brain, providing evidence of direct engagement with these myelin-producing cells [100]. Further supporting this, a preclinical study in Sprague Dawley rats (albeit one using a morphine-regimen model, not a demyelination model) found ibogaine administration increased gene expression and protein translation of key myelin-associated proteins, myelin basic protein (MBP) and 2′,3′-cyclic nucleotide 3′-phosphodiesterase (CNPase), in the rodent brain, with protein upregulation becoming highly significant 72 h post-administration [53]. However, studies in MS models are still limited.

Psychedelic-induced upregulation of neurotrophic factors like BDNF and GDNF also support oligodendrocyte maturation and survival, which are crucial steps in the remyelination process. Furthermore, PSYs anti-inflammatory actions—particularly reductions in pro-inflammatory cytokines such as interferon-γ and TNF-α mediated by 5-HT2A receptor activation—may remove inhibitory signals on oligodendrocyte maturation, thus facilitating the natural repair process [17,101].

Another important mechanism is the indirect support of oligodendrocyte function through the preservation of axonal integrity. Axons that remain structurally intact or are protected from degeneration can subsequently serve as substrates for remyelination [102].

Thus, neuroprotective effects exerted by PSYs indirectly enhance the remyelination capacity of surviving axons. Finally, PSYs induce transient increases in neural network activity and brain plasticity, which may facilitate neuronal-oligodendrocyte signaling, further priming oligodendrocyte progenitors toward repair. Indeed, neuronal activity itself can serve as a signaling mechanism that attracts oligodendrocyte precursors and stimulates remyelination [8,17,103,104].

**Figure 1:**
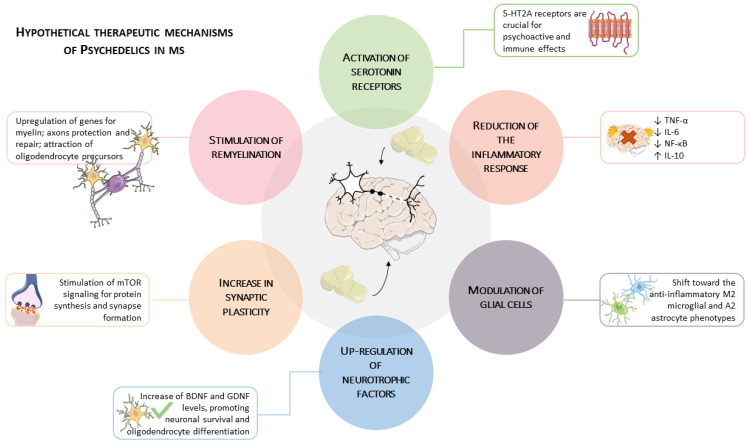
Proposed Multifactorial Therapeutic Mechanisms of Psychedelics (PSYs) in Multiple Sclerosis (MS). The model illustrates a dual-action potential. (Left & Top): PSYs activate serotonin receptors (e.g., 5-HT2A), leading to (1) a Reduction of the Inflammatory Response (e.g., ↓TNF-α, ↓IL-6) and (2) Modulation of Glial Cells toward anti-inflammatory phenotypes. (Right & Bottom): This activation also promotes (3) Upregulation of Neurotrophic Factors (e.g., BDNF), which drives (4) Increased Synaptic Plasticity and is hypothesized to support (5) Stimulation of Remyelination by aiding oligodendrocytes. This combination of anti-inflammatory and neuro-reparative actions is the central therapeutic hypothesis. The image was created using the image bank of Servier Medical Art (available online: http://smart.servier.com/, accessed on 20 September 2025), licensed under a Creative Commons Attribution 4.0 International License (available online: https://creativecommons.org/licenses/by/4.0/, accessed on 20 September 2025).

Direct evidence for PSY-induced remyelination in relevant animal models of MS is currently absent. The existing rationale is built upon a sparse collection of indirect and preliminary findings. These include an isolated case report of improved myelin markers in a patient treated with the atypical psychedelic ibogaine [20] and a preclinical study showing that psilocybin administration activates the immediate early gene *Fos* in oligodendrocytes [100]. When combined with the broader, albeit indirect, evidence of neurotrophic factor upregulation and immunomodulation, these observations allow for the formulation of a plausible, yet highly speculative, hypothesis regarding the pro-remyelinating potential of PSYs. Testing this hypothesis will require direct investigation in established toxic (e.g., cuprizone) and autoimmune (e.g., EAE) models of demyelination, which represents a critical next step for the field as summarized in Figure 1.

### 4.4. An Integrative Model: Synergies in Neuro-Reparative Pathways

The mechanisms discussed immunomodulation, synaptic plasticity, and oligodendrocyte support should not be viewed as entirely separate phenomena. We propose they likely interact synergistically. For example, the potent anti-inflammatory effects (Section 4.1) that shift microglia to a reparative M2 phenotype may be a direct prerequisite for effective remyelination (Section 4.3), as pro-inflammatory M1 microglia are known to inhibit oligodendrocyte progenitor cell (OPC) differentiation. Concurrently, the upregulation of neurotrophic factors like BDNF (Section 4.2) not only drives synaptic plasticity but also acts as a crucial survival signal for both mature neurons and oligodendrocytes. Thus, PSYs may establish a pro-reparative milieu where reduced inflammation and increased neurotrophic support converge to facilitate endogenous repair, a hypothesis that future EAE studies must explicitly test.

## 5. Challenges in Translating PSYs to MS Therapy

While Section 4 outlined the hypothetical mechanisms derived largely from non-MS-specific models, it is precisely this current scarcity of direct evidence that mandates a proactive analysis of translational barriers. Before resource-intensive EAE models or clinical trials are initiated, it is critical to assess whether the well-documented safety, legal, and logistical challenges of PSYs would render them non-viable for the unique MS patient population, even if efficacy were eventually proven. This ‘viability-first’ analysis is not premature; rather, it is essential for responsibly guiding future research and avoiding translational dead ends. This section therefore critically evaluates these barriers, which must be considered parallel to, not subsequent to, basic mechanistic research.

### 5.1. Safety and Neuropsychiatric Considerations

Despite the growing interest in the use of PSYs in MS, their clinical integration requires rigorous assessment of cardiovascular, neuropsychiatric, and pharmacological risks, which are particularly relevant in a population with complex comorbidities and multifocal neurological lesions.

#### 5.1.1. Cardiovascular Risks and Systemic Toxicity

Ibogaine, despite showing potential effects on remyelination in preclinical models [20,53], is associated with dose-dependent cardiotoxicity. In rat models, administration induces non-inflammatory myocardial necrosis [54]. At the clinical level, cases of cardiac arrest have been reported even at doses as low as 2.6 mg/kg [61], confirming an extremely narrow therapeutic margin. In vitro studies show that therapeutic ibogaine concentration (3 μM) significantly delays repolarization and prolongs the action potential in human induced pluripotent stem cell–derived cardiomyocytes [55]. Even classic PSYs such as psilocybin and LSD, despite presenting a more favorable acute toxicity profile [7,38,105], induce transient increases in systolic blood pressure and heart rate during the peak of the psychedelic effect. A meta-analysis of 30 studies has confirmed that these increases, although transient and manageable in controlled settings, occur across all dose ranges [106]. These changes, generally well tolerated in healthy individuals, may prove problematic in MS patients, where cardiovascular comorbidities are more prevalent compared to the general population [107,108]. This is particularly salient for the MS population, where chronic inflammation, reduced mobility, and certain disease-modifying therapies can independently contribute to cardiovascular risk, potentially lowering the threshold for adverse events. Furthermore, MS-related autonomic dysfunction could theoretically exacerbate psychedelic-induced cardiovascular events, such as arrhythmia or labile hypertension, a specific vulnerability does not present in healthy volunteer cohorts. Beyond these acute effects, recent reviews highlight the need to consider long-term risks, including arrhythmia, ischemia, and particularly valvular heart disease (VHD) [109]. The risk of VHD and cardiac fibrosis is a specific concern for chronic or microdosing regimens due to agonist activity at the serotonin 5-HT2B receptor, a mechanism shared with known cardiotoxic drugs [110,111]. The interaction between these physiological effects and pre-existing medical vulnerabilities is critical. For example, in patients with anorexia nervosa, psilocybin-induced tachycardia could be particularly risky in the context of potential myocardial atrophy, and any mild QT interval prolongation could become significant in the presence of electrolyte abnormalities. This underscores the necessity of thorough risk mitigation strategies, including baseline cardiovascular screening with ECG, correction of electrolyte imbalances prior to dosing, and continuous vital sign monitoring during sessions [112].

#### 5.1.2. Neuropsychiatric Vulnerability in MS

MS is associated with a high prevalence of psychiatric symptoms; the prevalence of Major Depressive Disorder (MDD) among individuals with MS can exceed 50%, while the prevalence of anxiety is approximately 22% [113,114].

In this context, the administration of psychedelic substances requires caution, as the induced perceptual alterations may exacerbate pre-existing disorders. For MS patients, who often grapple with disease-related anxiety, grief over functional loss, and cognitive deficits, the psychedelic-induced dissolution of ego could be experienced as particularly destabilizing if not expertly managed within a supportive therapeutic framework.

Analyses of naturalistic and clinical data indicate that this risk is disproportionately higher in vulnerable subpopulations. For instance, individuals with a prior diagnosis of a personality disorder show a more than four-fold elevated risk of adverse psychological responses after psychedelic use [115].

Specific long-term risks include Hallucinogen Persisting Perception Disorder (HPPD); one large survey found that 1.3% of lifetime psychedelic users reported receiving a formal HPPD diagnosis from a medical professional [116]. Furthermore, the “epistemic risk” has been identified, which is the potential for PSYs to induce radical shifts in a person’s core beliefs by altering the very criteria they use to validate reality, sometimes leading to the adoption of unfounded paranormal or metaphysical beliefs, subsequent social conflict, and epistemic isolation. On a mechanistic level, the “Psychedelic Iatrogenic Structural Dissociation” (PISD) hypothesis proposes that these substances may lower the defensive barriers between the part of the personality that handles daily life (the “Apparently Normal Personality”) and the part that holds traumatic memories (the “Emotional Personality”), leading to psychological destabilization, emotional dysregulation, and identity fragmentation in individuals with histories of trauma [117,118,119]. Ketamine, despite having a different receptor profile, is also not without psychiatric risks. Cases of persistent psychosis have been documented, especially in chronic users or in conjunction with other substances like cannabis [120]. It has also been observed that patients with ketamine-induced persistent psychosis show elevated levels of neurofilament light chain (NfL), a marker of neuroaxonal damage, higher than those found in patients with schizophrenia, indicating potential neurotoxicity associated with chronic use [67]. These risks, combined with its abuse potential [121], complicate its long-term use. However, it is noteworthy that the psychoactive effects of ketamine, such as mystical-type experiences, appear to mediate its therapeutic benefits in other contexts, such as alcohol dependence, highlighting the complex relationship between subjective experience and clinical outcome [68].

The unpredictability of subjective responses, modulated by individual and environmental factors, represents an additional critical issue. Patients with severe physical disabilities or disease-related anxieties might experience the psychedelic experience in a distorted or traumatic manner, including due to logistical difficulties in positioning during prolonged sessions (6–8 h) or the possibility of acute physical discomfort (e.g., spasticity) occurring during treatment.

These elements suggest the necessity of a rigorous selection of patients, with exclusion of subjects with a history of psychosis or bipolar disorders. This exclusion is strongly supported by survey data showing that 32.2% of individuals with bipolar disorder reported new or worsening symptoms, particularly mania, following psilocybin use [122]. Furthermore, the risk of severe adverse reactions is not confined to populations with known vulnerabilities. Recent case reports describe an enduring psychotic episode, accompanied by significant violence, after Ayahuasca use, leading to forensic psychiatric admission in a patient with no prior psychiatric risk factors [65]. Similarly, chronic MDMA use has been linked to severe treatment-refractory psychosis with catatonic features, particularly in individuals with a history of trauma, which failed to respond to conventional antipsychotics and required electroconvulsive therapy (ECT) for stabilization [66]. These cases underscore the potential for severe and unpredictable psychiatric outcomes. However, for context, a meta-analysis on depression trials showed that while a minority of participants (~10%) in the psilocybin arm experienced symptom worsening, this risk was no greater than that observed with an active comparator (escitalopram) and was lower than that of the waitlist control group.

#### 5.1.3. Pharmacological Interactions with DMTs

Given the serotonergic mechanism of action of classical PSYs such as psilocybin and LSD, their concomitant use with SSRIs, frequently prescribed in patients with depression, including those with MS may increase the risk of serotonin syndrome, especially in uncontrolled settings [15,77,123]. A critical area of pharmacological risk involves seizures; while classic PSYs alone do not appear to increase seizure risk in healthy individuals, analysis of online reports has highlighted that concomitant use with other substances, particularly lithium, is associated with a higher incidence of seizures [124]. Furthermore, the clinical management of interactions with corticosteroids, interferons, natalizumab, or fingolimod remains poorly defined [105,125,126,127].

The requirement for multidisciplinary assessment (including neurologists, psychiatrists, and cardiologists) and continuous monitoring fundamentally distinguishes these approaches from standard disease-modifying therapies (DMTs), which can often be administered without intensive supervision. Specifically, the use of ibogaine, due to its pro-arrhythmic properties and prolonged active half-life, necessitates administration protocols with continuous electrocardiographic monitoring and immediate clinical support [61]. Moreover, the extensive polypharmacy common in MS presents a significant hurdle. Potential pharmacokinetic and pharmacodynamic interactions between PSYs and common disease-modifying therapies (DMTs)—such as interferons, fingolimod, natalizumab, or ocrelizumab—are entirely uncharacterized and represent a critical translational knowledge gap. A consolidated overview of these cardiovascular, neuropsychiatric, and pharmacological risks is provided in Table 2.

### 5.2. Legal, Ethical, and Logistical Barriers

Many classic PSYs are classified as Schedule I controlled substances in numerous countries, denoting that they are illegal to possess and use outside of approved research. While recent years have seen a shift, with psilocybin gaining U.S. FDA “Breakthrough Therapy” designation for depression to speed development, they are not yet authorized for general medical use [128,129]. This cultural and scientific shift is mirrored in public perception; data from the U.S. National Survey on Drug Use and Health showed a significant linear decrease in the perceived risk of trying LSD between 2015 and 2019, although a majority of the public still considers it a high-risk activity [130]. This legal landscape is in constant flux; for example, states like Oregon and Colorado have created legal pathways for the therapeutic or supervised use of psilocybin, creating a complex tension between state and federal laws [131]. This discrepancy raises novel legal challenges, such as the lack of employment protections for patients who use psilocybin therapeutic in compliance with state law but in violation of federal law and workplace policies [132]. Furthermore, as commercial interest grows, the field is facing new legal hurdles related to intellectual property, with some entities attempting to patent psychedelic compounds and therapeutic protocols. This is complicated by inaccessible prior art from historical and unconventional sources and aggressive strategies like “patent thicketing,” which could threaten to privatize public domain knowledge and limit affordable access to these treatments [133,134].

Conducting clinical trials in MS will require rigorous regulations and approvals that acknowledge the controlled status of these drugs. This can slow down research and deter funding, as obtaining and administering Schedule I substances involves significant paperwork, oversight, and often stigma.

Ethically, some patients or providers might be hesitant to use a “psychedelic drug” due to lingering associations with 1960s counterculture or fears of unknown long-term effects [135,136].

Recent surveys of healthcare professionals confirm this gap: while many see therapeutic promise, they also report low objective knowledge about risks and pharmacology and express concerns about the lack of trained providers and potential contraindications [137]. This is mirrored by patients, many of whom do not disclose their psychedelic use to their physicians due to fears of stigma and perceived provider knowledge gaps [138,139]. To address these issues, experts are calling for structured ethical frameworks to guide the field. These include principles like the “Access, Reciprocity, and Conduct (ARC)” framework, which emphasizes equitable access, respect for traditional and Indigenous knowledge, and safe clinical conduct, as well as the integration of ethical principles from Indigenous traditions. A recent Indigenous-led consensus statement outlined eight core principles for this engagement: Reverence, Respect, Responsibility, Relevance, Regulation, Reparation, Restoration, and Reconciliation, urging the modern psychedelic movement to move beyond cultural appropriation and address tangible harms like unsustainable foraging and exploitative spiritual tourism [140,141].

Additionally, it should be noted that the model of care for psychedelic therapy (at least in mental health) involves extensive patient preparation, supervised drug session, and follow-up integration therapy [142,143]. For an MS patient, especially one with physical disabilities, attending a clinic for a full-day psychedelic session might be burdensome. Mobility issues, fatigue, and the need for assistive devices must be accommodated in the session environment. The unique needs of individuals with physical and sensory disabilities have historically been neglected in psychedelic research, risking the reinforcement of structural ableism in healthcare. There is an urgent need for research protocols to be designed inclusively, with extensive disability awareness training for therapists and appropriate accommodations [144]. The presence of neurological symptoms (e.g., spasticity or bladder dysfunction) could interrupt or complicate a session, for instance, needing to pause a psychedelic session to use the restroom, which can be challenging if the patient is experiencing visual distortions or impaired coordination. Logistical challenges are magnified in the MS population. A standard 6–8 h session may be prohibitive for patients dealing with significant fatigue, spasticity, or urgent bladder needs, requiring specialized clinical settings with staff trained not only in psychedelic therapy but also in neurological care.

Such practical issues mean that specialized centers and trained personnel would be required to administer psychedelic treatments to MS patients, at least initially. A significant logistical and ethical barrier is the training and conduct of therapists themselves. A major debate is whether personal experience with PSYs should be a prerequisite for therapists, with some experts arguing it is essential for empathy and understanding the client’s state, while others caution it is not sufficient for ensuring competence and may introduce bias [145]. This issue is further complicated by the risk of serious misconduct. The patient’s heightened vulnerability during psychedelic states creates a power imbalance that can be exploited, with documented cases of therapist abuse, including sexual misconduct [146]. The use of supportive physical touch, while considered by many practitioners to be a crucial component of therapy, is an area of intense debate and requires clear, consent-based protocols to avoid impropriety [147].

These necessities are distant from the current MS therapy model where patients self-administer injections at home or visit infusion centers briefly. Additionally, the resource intensity and cost of psychedelic-assisted therapy could be a major hurdle in justifying it versus existing treatments, unless future studies clearly demonstrate superior outcomes.

The rise of a for-profit psychedelic industry introduces further ethical challenges, including the risk that financial incentives and the commercial determinants of health could shift research agendas away from public need and compromise patient welfare for commercial gain [133,147,148]. Unlike depression or PTSD where typically one to three psychedelic sessions may suffice for a long-term effect [149], in MS a periodic dosing (e.g., monthly or quarterly) may be needed to continually suppress inflammation and/or promote repair. Chronic or repeated use of PSYs raises unanswered questions: Could tolerance to the psychological effects develop (perhaps beneficially allowing higher doses with less hallucination), or conversely might there be tachyphylaxis to the anti-inflammatory effects? There is some evidence from preclinical research that repeated low-dose (so-called “microdosing”) regimens can maintain psychoplastogenic effects without causing full psychedelic experiences [15,17]. If proven effective, such a model could be more cost-effective and scalable than high-dose sessions that require intensive psychotherapy [150]. Nevertheless, further studies would be needed to validate these observations. Designing trials to test microdosing in MS will be complex, and ensuring compliance (since even microdoses, still being Schedule I substances, would have to be managed closely) is non-trivial. Real-world data from new, regulated markets will be crucial for developing standardized protocols and monitoring safety and quality [151,152].

In summary, the path to clinical translation of PSYs for MS is fraught with challenges that extend beyond biological efficacy. Safety monitoring, patient acceptance, regulatory approval, and practical treatment delivery all pose significant hurdles. Any future clinical program will need to carefully address these through rigorous trial design, likely starting with small pilot studies to establish basic safety and feasibility in the MS population. It may also require creative solutions, such as developing non-hallucinogenic analogues or optimized dosing schedules, to make the therapy more palatable and scalable. These challenges are substantial but not insurmountable. They represent necessary caution on the road to innovating MS treatment.

## 6. Prospects for Clinical Use and Future Directions

The potential use of PSYs for MS treatment is still largely theoretical, but with preliminary evidence potentially supporting their use. Indeed, the reported case studies of MS patients experiencing lesion reduction and functional improvement after psychedelic treatment (ibogaine) provide support that neuroimmune modulation and even repair might be achievable by PSYs [20]. Nevertheless, structured clinical trials would be needed for rigorous observations.

### 6.1. Early Evidence and Dosing Strategies

Before any clinical trial can be designed, a critical preclinical question to resolve is the optimal dosing paradigm. Unlike psychiatric applications, where a single high-dose administration is typically employed, chronic neuroimmune disorders such as MS may benefit from repeated exposure. One early prospect to explore is microdosing strategies. Microdosing refers to taking sub-perceptual doses of a psychedelic repeatedly (e.g., roughly one-twentieth of a typical psychoactive dose, given a few times a week). Interestingly, some individuals with autoimmune conditions have experimented with microdosing to manage physiological symptoms, reporting benefits such as reduced migraines or improved energy [43,153,154,155]. Additionally, studies in murine models indicate that a single low dose of a psychedelic [R)-DOI] can induce anti-inflammatory gene expression (e.g., reducing TNF-α) without the acute behavioral effects that a large dose might cause [43,153]. A carefully controlled trial of microdosed psilocybin in MS could assess whether this regimen leads to any improvement in immune markers or patient-reported outcomes. If successful, such low-dose or sub-perceptual regimens could present a more practical treatment modality, one that might avoid the need for intense hallucinatory sessions and could potentially be self-administered under periodic supervision. It must be noted, however, that the clinical efficacy of microdosing remains unproven in any indication, and placebo-controlled studies are needed to validate any benefits.

### 6.2. Methodologies for Future Research: Biomarkers and Neuroimaging

Future preclinical studies in EAE models should incorporate a robust panel of biomarkers, such as neurofilament light chain (NfL) and glial fibrillary acidic protein (GFAP), to assess neuroaxonal damage and astrogliosis alongside clinical scores. This includes monitoring biofluid biomarkers such as neurofilament light chain (NfL), a validated indicator of neuroaxonal damage, glial fibrillary acidic protein (GFAP) as a marker of astroglial activation and circulating brain-derived neurotrophic factor (BDNF), which may reflect neuroplasticity. The direct relevance of these interconnected markers is underscored by preclinical evidence showing that MDMA-induced cytoskeletal damage causes a reduction in intracellular NfL, an effect that is partially rescued by the neuroprotective action of BDNF, thus providing a mechanistic link between the neurotrophic and neurodegenerative pathways that can be monitored in clinical trials [156]. The importance of NfL as a safety marker is further highlighted by studies on chronic ketamine use, where it was found that patients who develop persistent psychosis show significantly higher NfL levels, indicating measurable neuroaxonal damage that could be monitored to assess the long-term safety of these therapies [67]. Parallel evaluation of immune cell dynamics, such as the frequency of regulatory T cells and anti-inflammatory macrophages, is also crucial, particularly as preclinical data suggest PSYs may enhance these immunoregulatory responses, though this remains to be validated in humans.

Neuroimaging represents a complementary domain of investigation. Advanced MRI techniques, for which recent progress has made them sensitive to remyelination, such as magnetization transfer ratio (MTR), could help determine whether a given therapy reduces acute lesion formation or, critically, promotes remyelination in chronic plaques. Demonstrating even partial remyelination in longstanding MS lesions would be a paradigm-shifting finding. This would mark a transition from symptomatic management to a truly regenerative approach, given that remyelination is a robust process that restores the myelin sheath, which is key for axonal survival and preventing progressive, irreversible disability [157].

Equally important will be the integration of patient-reported outcomes—capturing what is clinically meaningful in domains such as fatigue, cognitive function, and daily functioning—to comprehensively assess the translational impact of novel interventions in MS. Establishing how a treatment affects the way a patient feels and functions is crucial, as statistically significant outcomes may not always be clinically significant for those living with the disease [158].

### 6.3. Integration with Existing Therapies and Dual Benefits

Should efficacy in relevant preclinical models be established, a long-term prospect to investigate would be the integration of PSYs with existing MS treatment paradigms for a multifaceted approach. For example, a patient on an immunomodulatory drug (like an interferon or ocrelizumab) might receive an adjunct psychedelic session a few times a year aimed at promoting CNS repair and mental health, or PSYs could be used sequentially following autologous hematopoietic stem cell transplantation (AHSCT) [159] to potentially offer synergistic modulation of immune activity and neural repair. The immunotherapy would handle peripheral immune suppression, while the psychedelic could work within the CNS to reduce inflammation (via microglia) and stimulate regrowth. Because many MS drugs do not cross the blood-brain barrier effectively [160,161], a psychedelic which readily enters the brain, could fill the gap by working directly on resident CNS cells [162,163]. Combining therapies could also allow lowering the dosage, potentially improving overall safety. Nevertheless, combination approaches would require careful study to ensure no adverse interactions.

Another area of integration is rehabilitation and psychosocial support. In another context, psilocybin, administered post-stroke, improves motor recovery, reduces neuroinflammation, and promotes neuroplasticity by activating the TrkB receptor and increasing levels of BDNF, MAP2, and synaptophysin. These findings suggest a therapeutic potential in post-stroke recovery [56]. In MS, where neurorehabilitation is crucial for regaining function lost after relapses, a similar approach could be tested. For instance, a patient could receive a moderate-dose psychedelic in a controlled setting and, once the acute hallucinations subside, engage in cognitive rehabilitation targeting memory and language, or even non-invasive neuromodulation techniques to enhance functional outcomes by engaging neuroplastic circuits at multiple levels. The psychedelic’s afterglow period, characterized by emotional openness, improved mood, and enhanced verbal fluency, might also help patients adopt healthier behaviors (like exercise or stress reduction techniques) that benefit long-term disease management [63,164]. Moreover, the documented psychological benefits of many PSYs could address comorbidities common in MS, like depression and anxiety, fostering psychological resilience alongside physical stabilization [7,8,165].

### 6.4. Development of Novel Psychedelic-Derived Therapies

Looking ahead, the ultimate therapeutic products may not be the classic PSYs themselves, but next-generation derivatives. The aim is to isolate or enhance beneficial properties like neuroimmune modulation and plasticity while minimizing the hallucinogenic effects. Efforts are already underway to create such “non-hallucinogenic psychoplastogens.” For instance, analogs of ibogaine, such as the non-toxic and non-hallucinogenic Tabernanthalog (TBG), and other 5-HT_2_A-biased partial agonists are being developed as ‘psychoplastogens’. This research aims to retain the robust pro-neurotrophic and anti-addictive effects while decoupling them from the cardiac toxicity and intense psychoactive properties of compounds like ibogaine [19,34,149,166]. Similarly, researchers have explored chemical scaffolds derived from LSD that may modulate intracellular growth pathways without inducing strong psychedelic experiences. Notably, (R)-DOI, a phenethylamine agonist, has been shown to potently suppress TNF-α-induced inflammation at picomolar concentrations [41]. While evidence for a complete dissociation between anti-inflammatory effects and hallucination proxies is still limited, preliminary findings suggest a potential for functional selectivity at the 5-HT2A receptor [44,167]. If such compounds can be refined, they could represent a new class of non-hallucinogen therapies. Pro-drug strategies, slow-release formulations, and CNS-targeted delivery systems may further optimize therapeutic efficacy and safety [168,169]. Other promising lines of development include tapping into the gut-brain-immune axis, as PSYs like ayahuasca may alter gut microbiota composition [170], and targeting the sigma-1 receptor (Sig-1R), engaged by DMT, for its neuroprotective roles [171].

### 6.5. Roadmap for Clinical Translation: Challenges and Outstanding Questions

Clinical translation will also require overcoming significant regulatory and cultural obstacles. Many classical PSYs remain classified as Schedule I substances, limiting research access. Nonetheless, regulatory precedents such as the FDA’s designation of psilocybin and MDMA as breakthrough therapies in psychiatry—offer a viable path forward. Establishing regulatory frameworks tailored to the specific safety and monitoring needs of psychedelic compounds, along with public engagement efforts to destigmatize their use, will be critical in advancing these agents toward clinical integration.

**Figure 2:**
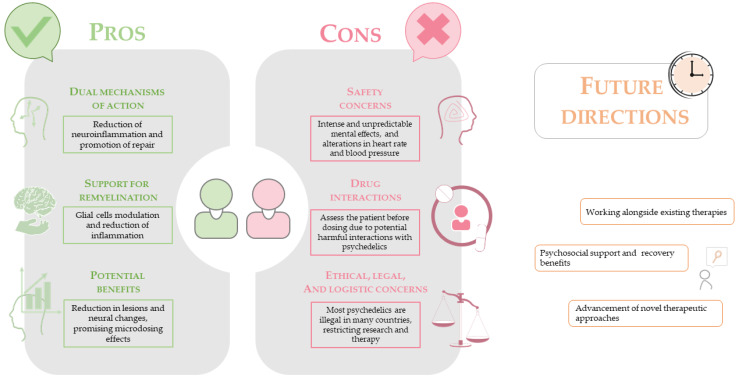
Summary of the Pros, Cons, and Future Directions of Psychedelics for Multiple Sclerosis Therapy. This infographic provides a balanced overview of the therapeutic landscape for psychedelic compounds (PSYs) in the context of MS. Pros (Left Panel): This section highlights the therapeutic potential, emphasizing the dual mechanisms of action where PSYs may simultaneously reduce neuroinflammation and promote neural repair. It underscores their potential support for remyelination, driven by the modulation of glial cells and the reduction of inflammatory signals that inhibit repair processes. The potential benefits also include observational evidence of lesion reduction and the promise of alternative dosing strategies like microdosing to achieve therapeutic effects. Cons (Center Panel): This section outlines the significant barriers to clinical translation. Safety concerns are paramount, including the intense and unpredictable psychoactive effects and the acute physiological stress, such as alterations in heart rate and blood pressure. The risk of adverse drug interactions with existing MS therapies and other medications necessitates thorough patient evaluation before administration. Finally, major ethical, legal, and logistical concerns exist, as most PSYs are classified as illegal controlled substances in many jurisdictions, which severely restricts research, funding, and therapeutic access. Future Directions (Right Panel): This section outlines the forward-looking strategies for the field. Key prospects include integrating PSYs as adjuncts working alongside existing therapies to potentially create synergistic effects on both immune suppression and CNS repair. Another promising avenue is leveraging the profound psychological effects of these compounds to provide psychosocial support and enhance recovery benefits, addressing comorbidities like depression and anxiety common in MS. The ultimate goal is the advancement of novel therapeutic approaches, such as developing non-hallucinogenic analogs that retain the beneficial immunomodulatory and neuroplastic properties while minimizing psychoactive side effects, offering a safer and more scalable treatment model. The image was created using the image bank of Servier Medical Art (available online: http://smart.servier.com/, accessed on 20 September 2025), licensed under a Creative Commons Attribution 4.0 International License (available online: https://creativecommons.org/licenses/by/4.0/, accessed on 20 September 2025).

Several outstanding questions remain and will guide the next generation of research. These include the determination of minimum effective dosing thresholds and the durability of therapeutic effects; the potential for synergy with remyelinating or neurorestorative agents; the identification of predictive biomarkers—such as neurophysiological or neurological connectomic signatures that correlate with response; and the influence of lesion burden or CNS microenvironment on the subjective and neurobiological effects of psychedelic treatment. Addressing these knowledge gaps will be essential in translating the scientific promise of PSYs into viable, evidence-based therapies for people living with MS (Figure 2).

## 7. Conclusions

The exploration of PSYs for MS represents an innovative frontier in neuroimmunology. Evidence indicates that classical PSYs and related compounds are multifaceted agents with the potential to concurrently attenuate neuroinflammation and stimulate neural repair.

Their primary mechanism involves the activation of serotonin receptors, which initiates downstream signaling that modulates cytokine profiles, reduces microglial activation, and promotes neuroplasticity and myelin regeneration. This dual immunomodulatory and neurorestorative action converges two therapeutic properties that are typically segregated in conventional MS treatments.

Despite promising mechanistic insights and preliminary clinical observations, significant challenges remain concerning the safety, legal status, and practical implementation of PSYs for MS. Rigorous controlled trials are imperative to establish a clear benefit-risk profile. The evolving regulatory landscape and positive outcomes in psychiatric research provide a favorable context for initiating such trials.

This research trajectory holds value beyond the direct clinical application of psychedelic compounds. Knowledge gained from these studies can illuminate novel biological pathways in MS pathology and guide the development of new, non-psychedelic therapeutics that harness these mechanisms without inducing psychoactive effects.

In addressing the practical question of which compounds represent the ‘least risk with most reward,’ a clear hierarchy emerges from the evidence reviewed. Compounds like ibogaine, despite intriguing preliminary data on myelin markers, likely represent a non-viable translational path due to a high and unpredictable risk of severe cardiotoxicity. Classical psychedelics, such as psilocybin, offer a more favorable acute safety profile and clear mechanistic relevance for both immunomodulation and neuroplasticity. However, their significant logistical, psychiatric, and regulatory challenges remain a primary barrier.

Therefore, this review concludes that the most pragmatically viable and scalable long-term strategy lies not with the classic compounds themselves, but with the next-generation non-hallucinogenic analogs they inspire. Functionally selective 5-HT2A agonists or ‘psychoplastogens’ like TBG designed to isolate the desired anti-inflammatory and neuro-reparative properties from the profound psychoactive effects—represent the most promising path forward for harnessing these mechanisms for MS therapy. Overall, the investigation of PSYs in MS is a significant interdisciplinary endeavor. It aligns with the contemporary therapeutic strategy of combining immune system recalibration with CNS regeneration. While the path to clinical translation is complex, the potential to pharmacologically induce a state of heightened plasticity and self-healing could fundamentally shift the MS treatment paradigm from managing neurodegeneration to enabling recovery and resilience.

## Figures and Tables

**Table 1:** Summary of preclinical and clinical evidence regarding psychedelic compounds and their potential relevance to MS. The studies are categorized into two main sections: Pre-Clinical models (in vitro and animal studies) and Clinical findings (human case reports and trials). This table summarizes the mechanisms of action, effects on neuroplasticity, inflammation, safety concerns, and potential for MS treatment of various compounds. It includes classical PSYs (such as LSD and psilocybin), ketamine, DMT, ibogaine, and synthetic analogs. The table highlights how each compound modulates neural plasticity, reduces neuroinflammation, and offers potential therapeutic benefits in MS, while also addressing safety concerns such as cardiotoxicity and psychiatric effects. Finally, the table underscores the research into synthetic analogs that aim to separate psychoplastogenic effects from the hallucinogenic burden, presenting them as a promising and safer alternative for MS treatment.

Substance	Model/Study Design	Key Mechanistic Finding	Relevant Outcome	Relevance for MS and Key Limitations	Ref.
Pre-Clinical Studies					
(R)-DOI	In vitro, RASMCs stimulated with TNF-α.	5-HT2A activation (confirmed with selective antagonists) inhibits nuclear translocation of NF-κB via a PKC-dependent pathway.	Suppression of pro-inflammatory gene expression (ICAM-1, VCAM-1, IL-6) and NOS activity with extraordinary potency (IC_50_ ~10–20 pM).	Relevance: Establishes the 5-HT2A/NF-κB mechanism and a picomolar potency, suggesting anti-inflammatory action is possible at sub-psychedelic doses. Limitations: In vitro model using vascular cells, not immune or CNS cells.	[41]
(R)-DOI	In vivo, murine model of systemic inflammation induced by TNF-α.	5-HT2A receptor activation (confirmed with the selective antagonist M100907).	Blockade of pro-inflammatory gene expression (Icam-1, Mcp-1, etc.) and circulating IL-6 at sub-behavioral doses. Effect is tissue-specific.	Relevance: Translates the potent anti-inflammatory effect to an animal model, confirming in vivo efficacy at non-psychotropic doses. Limitations: Acute induced-inflammation model, not a chronic autoimmune disease. The effect is tissue-specific, not global.	[42]
(R)-DOI	In vivo, mouse model of escapable social stress (Stress Alternatives Model-SAM).	Presumed 5-HT2A-mediated anti-inflammatory action.	A single low dose (0.015 mg/kg) promoted active coping strategies and reduced stress-induced TNF-α levels in plasma and limbic brain regions (BLA, PFC).	Relevance: Links anxiolytic/antidepressant-like behavioral effects with anti-inflammatory (TNF-α reduction) actions at sub-hallucinogenic doses in vivo. Limitations: Social stress model, not an autoimmune/demyelinating disease model.	[43]
(R)-DOI, (R)-DOTFM	In vivo, murine model of acute allergic asthma (OVA sensitization).	Demonstrates functional selectivity at the 5-HT2A receptor. The anti-inflammatory effect is linked to the suppression of arginase 1 (Arg1) expression.	(R)-DOI prevented airway inflammation, and this correlated with Arg1 suppression. The similar agonist (R)-DOTFM failed to suppress Arg1 and had no anti-inflammatory effect.	Relevance: Provides a specific molecular mechanism (Arg1 suppression) and highlights that not all 5-HT2A agonists are equal, crucial for designing new therapies. Limitations: Asthma is a peripheral, Th2-driven model, not a CNS autoimmune disease.	[44]
Various Psychedelic Analogs	In vivo, rat model of allergic asthma (OVA sensitization).	A systematic structure-activity relationship (SAR) study identified a common “anti-inflammatory pharmacophore” for 5-HT2A agonists distinct from the hallucinogenic pharmacophore.	The anti-inflammatory effect requires a specific chemical structure (e.g., 2,5-dimethoxy substitution) but is tolerant of changes at a different position (4-position) often associated with hallucinogenic potency.	Relevance: Identifies the 2C-H pharmacophore to decouple anti-inflammatory efficacy from hallucinogenic activity. Validates a chemical roadmap for non-psychotropic therapeutics. Limitations: Peripheral Th2-driven asthma model. Efficacy in neuroinflammatory conditions (e.g., MS) is theoretically inferred but not directly tested.	[45]
Psilocin	In vitro, murine (BV-2) and human (HL-60) microglial cell lines.	5-HT2 receptor activation (demonstrated using antagonists like cyproheptadine and risperidone).	Significant inhibition of reactive oxygen species (ROS), nitric oxide (NO) production, and phagocytic activity in LPS-activated microglia. No effect on TNF secretion.	Relevance: Provides direct evidence for a 5-HT2R-dependent mechanism by which psilocin modulates microglial effector functions (ROS, NO) central to neurodegenerative diseases and depression. Limitations: In vitro study on immortalized cell lines; requires validation in primary human microglia, human iPSC-derived brain organoids, and in vivo models.	[46]
Ketamine	In vivo, healthy rats (Forced Swimming Test - FST behavioral model).	Downstream activation of AMPA receptors, leading to increased mTOR phosphorylation and BDNF expression.	Rapid antidepressant-like effects (reduced immobility) associated with increased p-mTOR and BDNF levels in the hippocampus and prefrontal cortex.	Relevance: Elucidates the signaling cascade (AMPA→mTOR/BDNF) driving pro-plastic effects, a key mechanism for fast-acting antidepressant effects. Limitations: Behavioral model in healthy animals (Forced Swimming Test), restricted to acute stress response rather than a chronic depression model	[10]
Ketamine	In vivo, rat model of PTSD (Single-Prolonged Stress).	S-Ketamine pre-treatment attenuated pro-inflammatory responses.	Reduced stress-induced increases in TNF-α and IL-1β levels in key brain regions (striatum, periaqueductal gray).	Relevance: Links ketamine’s anxiolytic effects directly to the reduction of pro-inflammatory cytokines within the CNS, supporting its potential as a neuro-immunomodulator. Limitations: Lack of specific microglia inhibitors to confirm causality. Only prophylactic administration was tested.	[29]
Ketamine	In vivo, mouse model of inflammation-induced depression (LPS/TNF-α).	Ketamine’s prophylactic effect is mediated by the inhibition of the NLRP3 inflammasome signaling pathway specifically in the ventral hippocampus.	Ketamine pre-treatment prevented depressive-like behaviors induced by inflammatory stressors through suppression of NLRP3 activation.	Relevance: Identifies the NLRP3 inflammasome in the ventral hippocampus as a key target for ketamine’s pro-resilience effects against inflammation Limitations: Study limited to acute inflammatory stressors rather than chronic stress models; efficacy is strictly dose-dependent	[30]
Ketamine	In vivo, mouse model of post-stroke depression.	S-Ketamine’s effects are mediated by activation of the BDNF/TrkB pathway and modulation of synaptic proteins (PSD-95, SYP).	S-Ketamine ameliorated depressive-like behaviors, attenuated neuroinflammation (reduced Iba1, GFAP), and restored dendritic spine density post-stroke.	Relevance: Demonstrates that ketamine can promote synaptic restoration and reduce glial activation in a CNS injury model, providing strong evidence for its dual neuro-restorative and anti-inflammatory action. Limitations: Stroke is an acute ischemic injury, different from the chronic autoimmune demyelination of MS.	[31]
Ketamine	In vivo (mice) and in vitro (primary neurons), studies on antidepressant mechanisms.	IGF-1, in addition to BDNF, is an essential mediator for the rapid and sustained antidepressant effects of ketamine.	Ketamine increased extracellular IGF-1 levels in the mPFC. Blocking IGF-1 signaling (with a neutralizing antibody) abolished both the long-term and the immediate, antidepressant-like behavioral effects.	Relevance: Refines the model of ketamine’s action, showing that multiple neurotrophic factors (BDNF and IGF-1 for both rapid and sustained effects) are involved, which may be relevant for long-term repair in MS. Limitations: Study mainly in healthy animals, but confirmed in a disease model (LPS). Conducted only in male mice.	[47]
Ketamine	In vitro, human hippocampal progenitor cells (HPCs) treated with the pro-inflammatory cytokine IL-1β.	Ketamine prevents the inflammation-induced shift in the kynurenine pathway, reducing the production of the neurotoxic metabolite kynurenine (KYN)	IL-1β reduced neurogenesis and increased KYN production in HPCs. Co-treatment with ketamine rescued neurogenesis and normalized the kynurenine pathway.	Relevance: Provides a specific molecular mechanism linking inflammation to neurogenesis deficits and shows how ketamine can directly counter this, a highly relevant pathway for depression where inflammation impairs repair. Limitations: In vitro model using progenitor cells; needs in vivo validation in an depression model.	[28]
Psilocybin	In vivo (mice), chronic 2-photon microscopy of medial frontal cortex.	Structural remodeling (increased spine formation rate) is associated with 5-HT2AR activation but persists despite partial antagonism with ketanserin.	Rapid (~24 h) and persistent (>1 month) increases in dendritic spine density (~10%) and size. Increased mEPSC frequency.	Relevance: Provides direct in vivo evidence for rapid and long-lasting structural plasticity in the cortex, supporting a potential mechanism for sustained CNS repair. Limitations: Study in healthy animals, not a disease model. The exact contribution of 5-HT2AR vs. other targets remains debatable.	[48]
LSD, Psilocin	In vivo (mutant mice) and in vitro (HEK293T cells, neuronal cultures), biochemical/biophysical binding assays.	Proposed direct, high-affinity binding to the transmembrane domain of the BDNF receptor, TrkB.	Pro-plasticity and antidepressant-like effects are TrkB-dependent and 5-HT2A-independent. Hallucinogenic-like effects are 5-HT2A-dependent and TrkB-independent.	Relevance: Paradigm-shifting finding suggesting a direct link to the TrkB repair pathway, potentially separating therapeutic plasticity from hallucinosis. Limitations: Novel and debated mechanism that requires independent replication. The precise interplay between TrkB and 5-HT2A in vivo is still unclear.	[25]
Tabernanthalog (TBG)	In vitro (rat cortical neurons) and in vivo (rodent models of addiction/depression).	Designed as a 5-HT2A agonist that promotes structural plasticity (dendritic growth) in a ketanserin-sensitive manner.	Promotes neuroplasticity without inducing hallucinogenic-like effects (HTR). Shows antidepressant and anti-addictive effects with an improved safety profile (low cardiotoxicity) compared to ibogaine.	Relevance: Proof-of-concept for “non-hallucinogenic psychoplastogens,” a key future direction for developing tolerable MS therapies. Limitations: Neuroinflammatory effects are uncharacterized. Not tested in demyelination or autoimmunity models.	[49]
Psilocybin	In vivo, C57BL/6J mice; LPS-induced acute liver inflammation (pre- and post-treatment).	Inhibits macrophage pro-inflammatory cascade; specifically downregulates IL-12p70 protein (a key driver of Th1 and cytotoxic T-cell responses).	mRNA: Significant downregulation of IL-1β, IL-6, TNF-α, and COX-2 2. Protein: Significant reduction of IL-12p70 (Th1 driver), unlike other cytokines where changes were non-significant Histology: Reversed nuclear circularity defects (marker of cellular stress/apoptosis) and reduced inflammatory infiltration4	Relevance: IL-12p70 inhibition blocks Th1 differentiation, a critical pathway in MS autoimmunity. Limitations: Peripheral data (liver tissue), not CNS; acute inflammation model (not EAE/demyelinating).	[50]
DMT, Psilocin, 5-HT	In vitro (rat cortical neurons) and in vivo (mice with virally-expressed SERT in PFC).	Lipophilicity (membrane permeability) allows psychedelics to activate an intracellular pool of 5-HT2ARs, which mediates plasticity.	Lipophilic psychedelics (DMT, psilocin) promote plasticity; polar serotonin does not, unless given intracellular access (e.g., via SERT expression).	Relevance: Provides a fundamental explanation (location bias) for why psychedelics are effective psychoplastogens while endogenous serotonin is not. Limitations: Primarily an in vitro mechanistic study; the in vivo component uses an artificial system. Relevance in a disease state is unknown.	[51]
DMT	In vitro, human iPSC-derived cortical neurons and monocyte-derived microglia-like cells under hypoxia.	Activation of the Sigma-1 Receptor (Sig-1R), an intracellular chaperone protein.	Increased cell survival under severe hypoxic stress (0.5% O_2_). This protective effect was associated with downregulation of the hypoxia-inducible factor (HIF-1α).	Relevance: Identifies a neuroprotective mechanism (Sig-1R) independent of 5-HT receptors that could be relevant for mitigating hypoxic/ischemic damage within MS lesions. Limitations: In vitro model only; relevance of this anti-hypoxic mechanism to MS pathophysiology requires in vivo validation.	[52]
Ibogaine	In vivo, Sprague Dawley rats with a 10-day escalating morphine regimen.	Multi-receptor modulation (opioid, NMDA). Effect is an interaction between prior morphine exposure and ibogaine administration.	Administration after repeated morphine exposure significantly upregulated myelin markers CNP and MBP at mRNA (24 h) and protein (72 h) levels in the internal capsule.	Relevance: Provides rare preclinical evidence that ibogaine can upregulate key myelin proteins (CNP, MBP), supporting its hypothetical pro-remyelination potential. Limitations: The effect was only significant following morphine pre-treatment. Not a demyelinating disease model. Severe cardiotoxicity limits translation.	[53]
Ibogaine	In vivo, healthy male and female Wistar rats given a single oral dose (1 or 20 mg/kg).	Ibogaine induces myocardial necrosis without inflammatory infiltrate. The mechanism’s link to oxidative stress markers was inconsistent.	Dose-dependent, non-inflammatory myocardial necrosis observed 6 h and 24 h post-treatment in both sexes.	Relevance: Provides direct histopathological evidence of ibogaine’s severe cardiotoxicity (myocardial necrosis), a critical barrier to its clinical use for MS. Limitations: Did not directly link necrosis to arrhythmia; leaves the precise molecular pathway open to question.	[54]
Ibogaine, Noribogaine	In vitro, human induced pluripotent stem cell-derived cardiomyocytes (hiPSC-CMs).	Inhibition of repolarizing hERG potassium channels.	Therapeutic concentrations (3 µM) of both ibogaine and its long-lasting metabolite noribogaine significantly delay repolarization and prolong the action potential duration.	Relevance: Provides direct experimental proof in human cardiomyocytes for the cellular mechanism that underpins QT interval prolongation and arrhythmia risk. Limitations: In vitro model; does not capture systemic effects in a whole organism.	[55]
Psilocybin	In vitro (rat cortical neurons, glutamate excitotoxicity) and in vivo (rat model of stroke-MCAO).	Neuroprotection is mediated by the BDNF/TrkB pathway, as effects were blocked by the TrkB inhibitor ANA12.	Reduced glutamate-induced neuronal loss in vitro. Pre- and post-stroke treatment reduced brain infarction, improved locomotor behavior, upregulated MAP2/synaptophysin, and reduced microglial activation (IBA1).	Relevance: First demonstration of psilocybin’s neuroprotective and anti-inflammatory effects in a relevant CNS injury model (stroke). Directly links the therapeutic effect to the BDNF pathway. Limitations: Stroke is an acute ischemic injury, different from the chronic autoimmune demyelination of MS.	[56]
Psilocybin	In vivo, healthy domestic pigs; autoradiography at 1 and 7 days post-injection.	Agonist stimulation of 5-HT2AR leads to downstream synaptogenesis and transient receptor downregulation.	Increased SV2A density (synaptic marker) in the hippocampus (+4.4% at 1 d, +9.2% at 7 d) and PFC (+6.1% at 7 d). Transient downregulation of 5-HT2AR density at 1 d, which normalized by 7 d.	Relevance: Provides robust evidence for psilocybin-induced synaptogenesis in a large animal model with a gyrencephalic brain, increasing translational confidence. Limitations: Study in healthy animals, not a disease model. SV2A is a presynaptic marker; post-synaptic changes were not measured.	[26]
Psilocin, Ketamine	In vitro, primary rat cortical neurons.	Psychedelics and ketamine act on synaptogenesis via 5-HT2AR and TrkB signaling pathways.	Psilocin and ketamine both induced a comparable, rapid increase in the number and size of PSD-95 clusters (a postsynaptic marker), indicating synaptogenesis.	Relevance: Directly compares psilocin and ketamine, showing they promote synaptogenesis with similar efficacy and reinforcing the “psychoplastogen” concept for CNS repair. Limitations: In vitro study on healthy neurons. Does not model a disease state or neuroinflammation.	[57]
Hot-water extracts of 4 Psilocybe and Panaeolus mushroom species (containing psilocybin)	In vitro, Human U937 macrophages induced with LPS (1 μg/mL^3^)	Significant inhibition of TNF-α, IL-1β, IL-6, and COX-2	Potent suppression of inflammation with high safety profile (increased cell viability).	Relevance: argets key cytokines (TNF-α, IL-1β) involved in MS, but data is specific to osteoarthritis/general inflammation. Limitations: Uses whole extracts, not pure psilocybin. Effects likely driven by synergistic compounds (flavonoids, saponins).	[58]
MDMA	In vivo, rats exposed to a severe foot shock stress (SEFL) model.	MDMA administration attenuated the stress-induced increase of IL-1β and rescued microglial reduction (IBA-1) in the dorsal hippocampus.	MDMA administration significantly attenuated stress-enhanced fear learning	Relevance: Demonstrates anti-neuroinflammatory effects (specifically IL-1β) supporting the hypothesis that these compounds attenuate fear memory via immunosuppression. Limitations: Study used only male rats	[59]
**Clinical Studies**					
Psilocybin	Human (Healthy Volunteers)Randomized, double-blind, placebo-controlled trial (N = 60)	Acute reduction in plasma TNF-α significantly correlated with reduced glutamate concentrations in the hippocampus. Glial Status: No significant changes in myo-inositol (marker of glial activation).	Acute: Rapid suppression of TNF-α. Persisting (7 days): Sustained reduction of IL-6 and CRP (C-Reactive Protein)	Relevance: Targets two core MS pathologies: systemic inflammation (IL-6/CRP are drivers of autoimmunity) and excitotoxicity (glutamate-mediated axonal damage). Limitations: Study performed in healthy subjects, not patients with active neuroinflammation. Lack of effect on glial marker (myo-inositol) may weaken the rationale for targeting microglial activation directly.	[60]
Ibogaine	Case report (n = 1 human male with opioid dependence).	Acquired long QT syndrome leading to torsade de pointes.	Multiple episodes of cardiac arrest requiring defibrillation after a single, low dose (2.6 mg/kg) of ibogaine. QTc interval was massively prolonged (636 ms).	Relevance: Clinically demonstrates that even low, supposedly “safe” doses of ibogaine can be lethal, highlighting an extremely narrow and unpredictable therapeutic window. Limitations: An n = 1 case report; cannot establish causality or incidence rates.	[61]
Ibogaine	Case report (n = 1 human male).	Acquired long QT syndrome leading to life-threatening ventricular arrhythmia.	Cardiac arrest after a very high dose (5.6 g, ~65–70 mg/kg) of ibogaine. QTc was massively prolonged, and recovery took 7 days.	Relevance: Clinically demonstrates the severe, dose-related cardiotoxic risk of ibogaine. The long recovery time underscores the danger of its long-lasting metabolite, noribogaine. Limitations: An n = 1 case report. Hypokalemia was a co-trigger.	[62]
LSD (low dose)	Randomized, double-blind, placebo-controlled crossover trial (n = 24 healthy volunteers).	Not directly tested; discusses potential 5-HT2A involvement.	24 h post-dose, LSD improved visuospatial memory (ROCF, OLMT) and phonological verbal fluency, but impaired cognitive flexibility (increased perseverative errors on WCST).	Relevance: Provides human evidence for sub-acute pro-cognitive effects (memory/language). However, it also highlights a significant negative effect on executive function (cognitive rigidity), a key translational challenge. Limitations: Healthy volunteers, not MS patients. Low dose (50 µg).	[63]
Psilocybin	Randomized, waiting list-controlled clinical trial (n = 24 patients with MDD).	Not a mechanistic study; focused on clinical efficacy.	Two sessions of psilocybin with psychotherapy produced large, rapid, and sustained antidepressant effects (~71% response, ~54% remission at week 4).	Relevance: Establishes psilocybin as a powerful therapeutic tool for depression, a major comorbidity in MS. Provides a clinical framework and safety data for psychedelic-assisted therapy. Limitations: Not in MS patients. Waiting list control does not account for expectancy effects. Small sample size.	[64]
MDMA	Randomized, placebo-controlled Phase 3 trial (n = 104 patients with moderate to severe PTSD).	Not a mechanistic study; focused on clinical efficacy. Proposed mechanisms include increased monoamine release and reduced fear response.	MDMA-assisted therapy significantly reduced PTSD symptoms and functional impairment compared to placebo with therapy. Generally well-tolerated.	Relevance: Provides a regulatory roadmap (Phase 3 trial) for bringing psychedelic-like compounds to market. Establishes a model for combining a psychoactive substance with therapy for a CNS disorder. Limitations: Not in MS patients. MDMA is an entactogen with different primary mechanisms than classic psychedelics. Blinding is a significant challenge.	[22]
Ayahuasca	Case report (n = 1 human male).	Not applicable (clinical observation).	First-episode, enduring psychosis with significant violence requiring forensic psychiatric admission after on three occasions Ayahuasca ceremony in a patient with no prior risk factors.	Relevance: Highlights the severe and unpredictable psychiatric risks of psychedelics, even in individuals without pre-existing vulnerabilities, reinforcing the need for rigorous screening. Limitations: An n = 1 case report; cannot establish causality or incidence rates. Composition of the brew was unknown.	[65]
MDMA (chronic use)	Case report (n = 1 human female).	Not applicable (clinical observation).	Severe, treatment-refractory psychosis with catatonic features that failed to respond to conventional antipsychotics and required electroconvulsive therapy (ECT).	Relevance: Demonstrates potential for severe, persistent psychiatric syndromes with chronic use, a key concern for any proposed long-term MS therapy. Limitations: An n = 1 case report from a chronic, recreational use scenario, which may not be representative of controlled therapeutic use.	[66]
Ketamine (chronic use)	Clinical study, comparing ketamine-dependent patients with persistent psychosis to patients with schizophrenia and healthy controls.	Not applicable (biomarker study).	Patients with ketamine-induced persistent psychosis had significantly higher levels of neurofilament light chain (NfL), a marker of neuroaxonal damage, than both schizophrenia patients and healthy controls.	Relevance: Provides crucial clinical evidence that chronic ketamine use can be associated with measurable neuroaxonal injury, raising significant safety concerns for its long-term application in MS. Limitations: Cross-sectional study in a substance use population; does not establish a causal link between ketamine and neurotoxicity.	[67]
Ketamine	Randomized, controlled trial (n = 40 alcohol-dependent adults).	Mediation by psychoactive effects (mystical-type experience)	The “mystical-type experience” (measured by the Hood Mysticism Scale).	Relevance: Provides clinical evidence supporting the hypothesis that subjective, psychoactive effects are critical for efficacy. Limitations: Secondary analysis.	[68]

RASMCs: primary rat aortic smooth muscle cells; TNF-α: Tumor Necrosis Factor-alpha; 5-HT2A: Serotonin Receptor 2A; NF-κB: Nuclear Factor kappa-light-chain-enhancer of activated B cells; PKC: Protein Kinase C; ICAM-1: Intercellular Adhesion Molecule-1; VCAM-1: Vascular Cell Adhesion Molecule-1; IL-6: Interleukin-6; NOS: Nitric Oxide Synthase; IC_50_: Half maximal inhibitory concentration; MS: Multiple Sclerosis; Ref.: Reference; CNS: Central Nervous System; Mcp-1: Monocyte Chemoattractant Protein-1; SAM: Stress Alternatives Model; BLA: Basolateral Amygdala; PFC: Prefrontal Cortex; (R)-DOTFM: (R)-2,5-dimethoxy-4-(trifluoromethyl)amphetamine; OVA: Ovalbumin; Arg1: Arginase 1; Th2: T helper 2; SAR: Structure-Activity Relationship; ROS: Reactive Oxygen Species; NO: Nitric Oxide; EAE: Experimental Autoimmune Encephalomyelitis; FST: Forced Swimming Test; AMPA: α-amino-3-hydroxy-5-methyl-4-isoxazolepropionic acid Receptor; mTOR: mammalian Target of Rapamycin; BDNF: Brain-Derived Neurotrophic Factor; PTSD: Post-Traumatic Stress Disorder; IL-1β: Interleukin-1 beta; LPS: Lipopolysaccharide; NLRP3: NLR Family Pyrin Domain Containing 3; TrkB: Tropomyosin receptor kinase B; PSD-95: Postsynaptic Density protein 95; SYP: Synaptophysin; Iba1: Ionized calcium-binding adapter molecule 1; GFAP: Glial Fibrillary Acidic Protein; IGF-1: Insulin-like Growth Factor 1; mPFC: medial Prefrontal Cortex; HPCs: Hippocampal Progenitor Cells; QUIN: Quinolinic Acid; mEPSC: miniature Excitatory Postsynaptic Current; TBG: Tabernanthalog; HTR: Head-Twitch Response; UMS: Unpredictable Mild Stress; DMT: Dimethyltryptamine; 5-HT: 5-Hydroxytryptamine (Serotonin); SERT: Serotonin Transporter; iPSC: induced Pluripotent Stem Cell; Sig-1R: Sigma-1 Receptor; HIF-1α: Hypoxia-Inducible Factor-1α; NMDA: N-Methyl-D-aspartate Receptor; CNP: 2′,3′-Cyclic-nucleotide 3′-phosphodiesterase; MBP: Myelin Basic Protein; hiPSC-CMs: human induced pluripotent stem cell-derived cardiomyocytes; hERG: human Ether-à-go-go-Related Gene; QTc: Corrected QT interval; SV2A: Synaptic Vesicle Glycoprotein 2A; ROCF: Rey-Osterrieth Complex Figure test; OLMT: Object Location Memory Task; WCST: Wisconsin Card Sorting Test; MDD: Major Depressive Disorder; MCAO: Middle Cerebral Artery Occlusion; MAP2: Microtubule-Associated Protein 2; MDMA: 3,4-Methylenedioxymethamphetamine; 5-HT2B: Serotonin Receptor 2B; moDCs: monocyte-derived dendritic cells; TLR: Toll-Like Receptor; Th1/Th17: T helper 1/T helper 17; ECT: Electroconvulsive Therapy; NfL: Neurofilament light chain; MEQ30: Mystical Experience Questionnaire 3. This table highlights several key patterns across the cited studies: (1) The potent anti-inflammatory mechanisms (e.g., NF-κB inhibition) of 5-HT2A agonists like (R)-DOI are often observed at sub-behavioral doses, suggesting a potential therapeutic window that separates immunomodulation from psychoactive effects. (2) Ketamine, while mechanistically distinct (NMDA antagonist), converges on the same core pro-plasticity pathways (e.g., BDNF/TrkB/mTOR) as classic psychedelics. (3) A critical translational filter is evident (e.g., ibogaine), where promising preliminary pro-myelin data is rendered clinically non-viable by severe cardiotoxicity, underscoring the field’s necessary pivot toward safer, non-hallucinogenic analog.

**Table 2:** This table summarizes the primary cardiovascular, neuropsychiatric, and other key risks associated with various psychedelic compounds. It highlights specific safety concerns such as cardiotoxicity, serotonin syndrome, and adverse psychiatric effects, which are critical for evaluating the translational feasibility of these substances for MS treatment.

PSYs	Cardiovascular Risks	Neuropsychiatric Risks	Other Risks	Ref
Ibogaine	High cardiotoxicity risk, dose-dependent myocardial necrosis, cardiac arrest, QT prolongation, arrhythmias.	Hallucinations	Requires continuous ECG monitoring.	[54]
LSD	Transient increase in blood pressure and heart rate. Chronic use: VHD risk (5-HT2B agonism).	May increase anxiety, HPPD, psychosis	Serotonergic toxicity, especially with SSRIs.	[15,105,110,116]
Psilocybin	Transient increase in blood pressure (systolic and diastolic) and heart rate.	Can worsen pre-existing psychiatric conditions, particularly bipolar disorder and psychosis.	Risk of serotonin syndrome, especially with concurrent use of other serotonergic medications.	[106,122]
Ketamine		Dissociation, hallucinations, abuse/addiction risk, persistent psychosis (NfL↑).	Bladder toxicity with chronic use	[67,68,121]
DMT (Ayahuasca)	Increases BP/HR, especially with MAOIs (Ayahuasca).	Intense hallucinations, risk of psychosis even without prior history.	Risk of serotonin syndrome (with SSRIs/MAOIs)	[39,65]
Analogs (e.g., DOI)	Activates 5-HT2A receptors, which can modulate vasoconstriction	Produces hallucinogenic effects at specific doses and is classified as a psychedelic.	At higher doses, risks include vasoconstriction. The drug’s mechanism, excessive mTOR stimulation, has been associated with other disorders	[34,41,42]

PSYs: Psychedelics; ECG: Electrocardiogram; LSD: Lysergic acid diethylamide; VHD: Valvular Heart Disease; 5-HT2B: 5-hydroxytryptamine receptor 2B; HPPD: Hallucinogen Persisting Perception Disorder; SSRIs: Selective Serotonin Reuptake Inhibitors; NfL: Neurofilament light chain; BP: Blood Pressure; HR: Heart Rate; DMT: N,N-Dimethyltryptamine; MAOIs: Monoamine oxidase inhibitor; DOI: 2,5-Dimethoxy-4-iodoamphetamine.

## Data Availability

No new data were created or analyzed in this study.

## Abbreviations

5-HT	5-hydroxytryptamine (Serotonin)
5-HT2A	5-hydroxytryptamine receptor 2A
5-MeO-DMT	5-methoxy-N,N-dimethyltryptamine
AHSCT	Autologous Hematopoietic Stem Cell Transplantation
AI	Artificial Intelligence
AMPA	α-amino-3-hydroxy-5-methyl-4-isoxazolepropionic acid receptor
Arg1	Arginase 1
Ayahuasca	Psychoactive brew containing DMT and MAOIs
BDNF	Brain-Derived Neurotrophic Factor
BP	Blood Pressure
CNS	Central Nervous System
CNP/CNPase	2′,3′-cyclic nucleotide 3′-phosphodiesterase
COX-2	Cyclooxygenase-2
CRP	C-reactive protein
DMF	Dimethyl fumarate
DMT	N,N-Dimethyltryptamine
DMTs	Disease-Modifying Therapies
DOI	(R)-2,5-Dimethoxy-4-iodoamphetamine
ECG	Electrocardiogram
ECT	Electroconvulsive Therapy
FDA	Food and Drug Administration
GDNF	Glial cell line-derived neurotrophic factor
GFAP	Glial Fibrillary Acidic Protein
GR	Glutathione reductase
hiPSCs	human induced Pluripotent Stem Cells
HPPD	Hallucinogen Persisting Perception Disorder
HR	Heart Rate
IL-6	Interleukin-6
iNOS	inducible nitric oxide synthase
i.p.	intra-peritoneal
Ketamine	2-(2-chlorophenyl)-2-(methylamino)cyclohexanone
LPS	Lipopolysaccharide
LSD	Lysergic acid diethylamide
MAOI	Monoamine oxidase inhibitor
MBP	Myelin basic protein
MDD	Major Depressive Disorder
MDMA	3,4-Methylenedioxymethamphetamine
moDC	Monocyte-derived Dendritic Cells
moMAC	Monocyte-derived Macrophages
MS	Multiple Sclerosis
mTOR	mammalian target of rapamycin
MTR	magnetization transfer ratio
NF-κB	nuclear factor kappa B
NfL	Neurofilament light chain
NKCC	Natural Killer (NK) cells
NMDA	N-methyl-D-aspartate
NOX	NADPH oxidase
PISD	Psychedelic Iatrogenic Structural Dissociation
Psilocybin	4-phosphoryloxy-N,N-dimethyltryptamine
PSYs	Psychedelics
PTSD	post-traumatic stress disorder
PV+	parvalbumin-positive
ROS	Reactive oxygen species
S1R	Sigma-1
SOD	superoxide dismutase 1
SSRIs	Selective Serotonin Reuptake Inhibitors
SV2A	synaptic vesicle protein 2A
TBG	Tabernanthalog
TNF-α	tumor necrosis factor-alpha
TrkB	Tropomyosin receptor kinase B
VHD	valvular heart disease
y/o	year-old

## References

[1] Kiss M.G., Mindur J.E., Yates A.G., Lee D., Fullard J.F., Anzai A., Poller W.C., Christie K.A., Iwamoto Y., Roudko V. (2023). Interleukin-3 Coordinates Glial-Peripheral Immune Crosstalk to Incite Multiple Sclerosis. Immunity.

[2] Yang J.H., Rempe T., Whitmire N., Dunn-Pirio A., Graves J.S. (2022). Therapeutic Advances in Multiple Sclerosis. Front. Neurol..

[3] Pozzilli C., Pugliatti M., Vermersch P., Grigoriadis N., Alkhawajah M., Airas L., Oreja-Guevara C. (2023). Diagnosis and Treatment of Progressive Multiple Sclerosis: A Position Paper. Eur. J. Neurol..

[4] Mansilla M.J., Presas-Rodríguez S., Teniente-Serra A., González-Larreategui I., Quirant-Sánchez B., Fondelli F., Djedovic N., Iwaszkiewicz-Grześ D., Chwojnicki K., Miljković Đ. (2021). Paving the Way towards an Effective Treatment for Multiple Sclerosis: Advances in Cell Therapy. Cell. Mol. Immunol..

[5] Auletta J.J., Bartholomew A.M., Maziarz R.T., Deans R.J., Miller R.H., Lazarus H.M., Cohen J.A. (2012). The Potential of Mesenchymal Stromal Cells as a Novel Cellular Therapy for Multiple Sclerosis. Immunotherapy.

[6] Szabo A. (2015). Psychedelics and Immunomodulation: Novel Approaches and Therapeutic Opportunities. Front. Immunol..

[7] Chi T., Gold J.A. (2020). A Review of Emerging Therapeutic Potential of Psychedelic Drugs in the Treatment of Psychiatric Illnesses. J. Neurol. Sci..

[8] de Vos C.M.H., Mason N.L., Kuypers K.P.C. (2021). Psychedelics and Neuroplasticity: A Systematic Review Unraveling the Biological Underpinnings of Psychedelics. Front. Psychiatry.

[9] Lima da Cruz R.V., Moulin T.C., Petiz L.L., Leão R.N. (2018). Corrigendum: A Single Dose of 5-MeO-DMT Stimulates Cell Proliferation, Neuronal Survivability, Morphological and Functional Changes in Adult Mice Ventral Dentate Gyrus. Front. Mol. Neurosci..

[10] Zhou W., Wang N., Yang C., Li X.M., Zhou Z.Q., Yang J.J. (2014). Ketamine-Induced Antidepressant Effects Are Associated with AMPA Receptors-Mediated Upregulation of MTOR and BDNF in Rat Hippocampus and Prefrontal Cortex. Eur. Psychiatry.

[11] Siegel A.N., Meshkat S., Benitah K., Lipsitz O., Gill H., Lui L.M.W., Teopiz K.M., McIntyre R.S., Rosenblat J.D. (2021). Registered Clinical Studies Investigating Psychedelic Drugs for Psychiatric Disorders. J. Psychiatr. Res..

[12] Aleksandrova L.R., Phillips A.G. (2021). Neuroplasticity as a Convergent Mechanism of Ketamine and Classical Psychedelics. Trends Pharmacol. Sci..

[13] Khan S.M., Carter G.T., Aggarwal S.K., Holland J. (2021). Psychedelics for Brain Injury: A Mini-Review. Front. Neurol..

[14] de Deus J.L., Maia J.M., Soriano R.N., Amorim M.R., Branco L.G.S. (2025). Psychedelics in neuroinflammation: Mechanisms and therapeutic potential. Prog. Neuropsychopharmacol. Biol. Psychiatry.

[15] Inserra A., De Gregorio D., Gobbi G. (2021). Psychedelics in Psychiatry: Neuroplastic, Immunomodulatory, and Neurotransmitter Mechanisms. Pharmacol. Rev..

[16] Weiss F., Magnesa A., Gambini M., Gurrieri R., Annuzzi E., Elefante C., Perugi G., Marazziti D. (2025). Psychedelic-Induced Neural Plasticity: A Comprehensive Review and a Discussion of Clinical Implications. Brain Sci..

[17] Kozlowska U., Nichols C., Wiatr K., Figiel M. (2022). From Psychiatry to Neurology: Psychedelics as Prospective Therapeutics for Neurodegenerative Disorders. J. Neurochem..

[18] Banks M.I., Zahid Z., Jones N.T., Sultan Z.W., Wenthur C.J. (2021). Catalysts for Change: The Cellular Neurobiology of Psychedelics. Mol. Biol. Cell.

[19] Winkelman M.J., Szabo A., Frecska E. (2023). The Potential of Psychedelics for the Treatment of Alzheimer’s Disease and Related Dementias. Eur. Neuropsychopharmacol..

[20] Chen D.Q., Inzunza Domínguez J.A., Valle Uzeta J.M., Pushparaj A.P., Dickinson J.E. (2025). Case Report: Significant Lesion Reduction and Neural Structural Changes Following Ibogaine Treatments for Multiple Sclerosis. Front. Immunol..

[21] Behera H.K., Joga R., Yerram S., Karnati P., Mergu T., Gandhi K., Sowndharya M., Nathiya D., Singh R.P., Srivastava S. (2024). Exploring the Regulatory Framework of Psychedelics in the US & Europe. Asian J. Psychiatr..

[22] Mitchell J.M., Ot’alora G M., van der Kolk B., Shannon S., Bogenschutz M., Gelfand Y., Paleos C., Nicholas C.R., Quevedo S., Balliett B. (2023). MDMA-Assisted Therapy for Moderate to Severe PTSD: A Randomized, Placebo-Controlled Phase 3 Trial. Nat. Med..

[23] Kim K., Che T., Panova O., DiBerto J.F., Lyu J., Krumm B.E., Wacker D., Robertson M.J., Seven A.B., Nichols D.E. (2020). Structure of a Hallucinogen-Activated Gq-Coupled 5-HT2A Serotonin Receptor. Cell.

[24] Zota I., Chanoumidou K., Gravanis A., Charalampopoulos I. (2024). Stimulating Myelin Restoration with BDNF: A Promising Therapeutic Approach for Alzheimer’s Disease. Front. Cell Neurosci..

[25] Moliner R., Girych M., Brunello C.A., Kovaleva V., Biojone C., Enkavi G., Antenucci L., Kot E.F., Goncharuk S.A., Kaurinkoski K. (2023). Psychedelics Promote Plasticity by Directly Binding to BDNF Receptor TrkB. Nat. Neurosci..

[26] Raval N.R., Johansen A., Donovan L.L., Ros N.F., Ozenne B., Hansen H.D., Knudsen G.M. (2021). A Single Dose of Psilocybin Increases Synaptic Density and Decreases 5-HT2A Receptor Density in the Pig Brain. Int. J. Mol. Sci..

[27] Heal D.J., Gosden J., Smith S.L., Atterwill C.K. (2023). Experimental Strategies to Discover and Develop the next Generation of Psychedelics and Entactogens as Medicines. Neuropharmacology.

[28] Mandal G., Kirkpatrick M., Alboni S., Mariani N., Pariante C.M., Borsini A. (2024). Ketamine Prevents Inflammation-Induced Reduction of Human Hippocampal Neurogenesis via Inhibiting the Production of Neurotoxic Metabolites of the Kynurenine Pathway. Int. J. Neuropsychopharmacol..

[29] Yang S., Xu K., Xu X., Zhu J., Jin Y., Liu Q., Xu R., Gu X., Liu Y., Huang Y. (2022). S-Ketamine Pretreatment Alleviates Anxiety-Like Behaviors and Mechanical Allodynia and Blocks the Pro-Inflammatory Response in Striatum and Periaqueductal Gray From a Post-Traumatic Stress Disorder Model. Front. Behav. Neurosci..

[30] Camargo A., Dalmagro A.P., Wolin I.A.V., Kaster M.P., Rodrigues A.L.S. (2021). The Resilient Phenotype Elicited by Ketamine against Inflammatory Stressors-Induced Depressive-like Behavior Is Associated with NLRP3-Driven Signaling Pathway. J. Psychiatr. Res..

[31] Tian J., Xie Y., Ye S., Hu Y., Feng J., Li Y., Lou Z., Ruan L., Wang Z. (2025). S-Ketamine Ameliorates Post-Stroke Depression in Mice via Attenuation of Neuroinflammation, Synaptic Restoration, and BDNF Pathway Activation. Biochem. Biophys. Res. Commun..

[32] Kubota M., Niwa H., Seya K., Kawaguchi J., Kushikata T., Hirota K. (2022). Ketamine Does Not Change Natural Killer Cell Cytotoxicity in Patients Undergoing Cancer Surgery: Basic Experiment and Clinical Trial. J. Oncol..

[33] Wu H., Savalia N.K., Kwan A.C. (2021). Ketamine for a Boost of Neural Plasticity: How, but Also When?. Biol. Psychiatry.

[34] Olson D.E. (2018). Psychoplastogens: A Promising Class of Plasticity-Promoting Neurotherapeutics. J. Exp. Neurosci..

[35] Wu W., Gong X., Qin Z., Wang Y. (2025). Molecular Mechanisms of Excitotoxicity and Their Relevance to the Pathogenesis of Neurodegenerative Diseases—An Update. Acta Pharmacol. Sin..

[36] Wang X., Chang L., Wan X., Tan Y., Qu Y., Shan J., Yang Y., Ma L., Hashimoto K. (2022). (R)-Ketamine Ameliorates Demyelination and Facilitates Remyelination in Cuprizone-Treated Mice: A Role of Gut–Microbiota–Brain Axis. Neurobiol. Dis..

[37] Lullau A.P.M., Haga E.M.W., Ronold E.H., Dwyer G.E. (2023). Antidepressant Mechanisms of Ketamine: A Review of Actions with Relevance to Treatment-Resistance and Neuroprogression. Front. Neurosci..

[38] Lutfy K., Pechnick R.N., Darmani N.A. (2023). Editorial: Pharmacology of New Psychoactive Substances. Front. Pharmacol..

[39] Egger K., Aicher H.D., Cumming P., Scheidegger M. (2024). Neurobiological Research on N,N-Dimethyltryptamine (DMT) and Its Potentiation by Monoamine Oxidase (MAO) Inhibition: From Ayahuasca to Synthetic Combinations of DMT and MAO Inhibitors. Cell. Mol. Life Sci..

[40] Deliganis A.V., Pierce P.A., Peroutka S.J. (1991). Differential Interactions of Dimethyltryptamine (DMT) with 5-HT1A and 5-HT2 Receptors. Biochem. Pharmacol..

[41] Yu B., Becnel J., Zerfaoui M., Rohatgi R., Boulares A.H., Nichols C.D. (2008). Serotonin 5-Hydroxytryptamine2A Receptor Activation Suppresses Tumor Necrosis Factor-α-Induced Inflammation with Extraordinary Potency. J. Pharmacol. Exp. Ther..

[42] Nau F., Yu B., Martin D., Nichols C.D. (2013). Serotonin 5-HT2A Receptor Activation Blocks TNF-α Mediated Inflammation In Vivo. PLoS ONE.

[43] Krupp K.T., Yaeger J.D.W., Ledesma L.J., Withanage M.H.H., Gale J.J., Howe C.B., Allen T.J., Sathyanesan M., Newton S.S., Summers C.H. (2024). Single Administration of a Psychedelic [(R)-DOI] Influences Coping Strategies to an Escapable Social Stress. Neuropharmacology.

[44] Flanagan T.W., Foster T.P., Galbato T.E., Lum P.Y., Louie B., Song G., Halberstadt A.L., Billac G.B., Nichols C.D. (2024). Sero-tonin-2 Receptor Agonists Produce Anti-Inflammatory Effects Through Functionally Selective Mechanisms That Involve the Suppression of Disease-Induced Arginase 1 Expression. ACS Pharmacol. Transl. Sci..

[45] Flanagan T.W., Billac G.B., Landry A.N., Sebastian M.N., Cormier S.A., Nichols C.D. (2021). Structure-Activity Relationship Analysis of Psychedelics in a Rat Model of Asthma Reveals the Anti-Inflammatory Pharmacophore. ACS Pharmacol. Transl. Sci..

[46] Wiens K.R., Brooks N.A.H., Riar I., Greuel B.K., Lindhout I.A., Klegeris A. (2024). Psilocin, the Psychoactive Metabolite of Psilocybin, Modulates Select Neuroimmune Functions of Microglial Cells in a 5-HT2 Receptor-Dependent Manner. Molecules.

[47] Deyama S., Kondo M., Shimada S., Kaneda K. (2022). IGF-1 Release in the Medial Prefrontal Cortex Mediates the Rapid and Sustained Antidepressant-like Actions of Ketamine. Transl. Psychiatry.

[48] Shao L.-X., Liao C., Gregg I., Davoudian P.A., Savalia N.K., Delagarza K., Kwan A.C. (2021). Psilocybin Induces Rapid and Persistent Growth of Dendritic Spines in Frontal Cortex In Vivo. Neuron.

[49] Cameron L.P., Tombari R.J., Lu J., Pell A.J., Hurley Z.Q., Ehinger Y., Vargas M.V., McCarroll M.N., Taylor J.C., Myers-Turnbull D. (2021). A Non-Hallucinogenic Psychedelic Analogue with Therapeutic Potential. Nature.

[50] Robinson G.I., Gerasymchuk M., Zanikov T., Gojani E.G., Asghari S., Groves A., Haselhorst L., Nandakumar S., Stahl C., Cruz C. (2025). LPS-Induced Liver Inflammation Is Inhibited by Psilocybin and Eugenol in Mice. Pharmaceuticals.

[51] Vargas M.V., Dunlap L.E., Dong C., Carter S.J., Tombari R.J., Jami S.A., Cameron L.P., Patel S.D., Hennessey J.J., Saeger H.N. (2023). Psychedelics Promote Neuroplasticity through the Activation of Intracellular 5-HT2A Receptors. Science.

[52] Szabo A., Kovacs A., Riba J., Djurovic S., Rajnavolgyi E., Frecska E. (2016). The Endogenous Hallucinogen and Trace Amine N,N-Dimethyltryptamine (DMT) Displays Potent Protective Effects Against Hypoxia via Sigma-1 Receptor Activation in Human Primary IPSC-Derived Cortical Neurons and Microglia-like Immune Cells. Front. Neurosci..

[53] Govender D., Moloko L., Papathanasopoulos M., Tumba N., Owen G., Calvey T. (2024). Ibogaine Administration Following Repeated Morphine Administration Upregulates Myelination Markers 2′, 3′-Cyclic Nucleotide 3′-Phosphodiesterase (CNP) and Myelin Basic Protein (MBP) MRNA and Protein Expression in the Internal Capsule of Sprague Dawley Rats. Front. Neurosci..

[54] Vidonja Uzelac T., Tatalović N., Mijović M., Miler M., Grahovac T., Oreščanin Dušić Z., Nikolić-Kokić A., Blagojević D. (2024). Ibogaine Induces Cardiotoxic Necrosis in Rats—The Role of Redox Processes. Int. J. Mol. Sci..

[55] Rubi L., Eckert D., Boehm S., Hilber K., Koenig X. (2017). Anti-Addiction Drug Ibogaine Prolongs the Action Potential in Human Induced Pluripotent Stem Cell-Derived Cardiomyocytes. Cardiovasc. Toxicol..

[56] Yu S.J., Wu K.J., Wang Y.S., Bae E., Chianelli F., Bambakidis N., Wang Y. (2024). Neuroprotective Effects of Psilocybin in a Rat Model of Stroke. BMC Neurosci..

[57] Vella Y., Syrová K., Petrušková A., Koutrouli I., Kútna V., Pala J., Šíchová K., Nikolič M., Mazoch V., Jurok R. (2025). Effects of Serotonergic Psychedelics on Synaptogenesis and Immediate Early Genes Expression—Comparison with Ketamine, Fluoxetine and Lithium. J. Psychopharmacol..

[58] Nkadimeng S.M., Steinmann C.M.L., Eloff J.N. (2021). Anti-Inflammatory Effects of Four Psilocybin-Containing Magic Mushroom Water Extracts In Vitro on 15-Lipoxygenase Activity and on Lipopolysaccharide-Induced Cyclooxygenase-2 and Inflammatory Cytokines in Human U937 Macrophage Cells. J. Inflamm. Res..

[59] Parekh S.V., Adams L.O., Barkell G.A., Lysle D.T. (2022). MDMA Administration Attenuates Hippocampal IL-β Immunoreactivity and Subsequent Stress-Enhanced Fear Learning: An Animal Model of PTSD. Brain Behav. Immun. Health.

[60] Mason N.L., Szabo A., Kuypers K.P.C., Mallaroni P.A., de la Torre Fornell R., Reckweg J.T., Tse D.H.Y., Hutten N.R.P.W., Feilding A., Ramaekers J.G. (2023). Psilocybin Induces Acute and Persisting Alterations in Immune Status in Healthy Volunteers: An Experimental, Placebo-Controlled Study. Brain Behav. Immun..

[61] Mestre D., Paula A., Gil F.P., Vaz J. (2024). Multiple Episodes of Cardiac Arrest Induced by Treatment with Ibogaine: A Case Report. Cureus.

[62] Steinberg C., Deyell M.W. (2018). Cardiac Arrest after Ibogaine Intoxication. J. Arrhythm..

[63] Wießner I., Olivieri R., Falchi M., Palhano-Fontes F., Oliveira Maia L., Feilding A., Araujo D.B., Ribeiro S., Tófoli L.F. (2022). LSD, Afterglow and Hangover: Increased Episodic Memory and Verbal Fluency, Decreased Cognitive Flexibility. Eur. Neuropsychopharmacol..

[64] Davis A.K., Barrett F.S., May D.G., Cosimano M.P., Sepeda N.D., Johnson M.W., Finan P.H., Griffiths R.R. (2021). Effects of Psilocybin-Assisted Therapy on Major Depressive Disorder: A Randomized Clinical Trial. JAMA Psychiatry.

[65] Baker M.R., O’Shea C.I. (2024). Drug-Induced Psychosis Following Use of Ayahuasca: A Presentation to Forensic Psychiatric Services. BMJ Case Rep..

[66] Litenski M.N., O’Reardon A.B., Pabon N., Hernandez M., Niyazov Y., Cruz J. (2024). Navigating Treatment Challenges: A Case Study on Refractory Psychosis in a Chronic MDMA (3,4-Methylenedioxymethamphetamine) User. Cureus.

[67] Chung A.N., Huang M.C., Liu T.H., Chang H.M., Chen P.Y., Liu Y.L., Bavato F. (2024). Ketamine-Dependent Patients with Persistent Psychosis Have Higher Neurofilament Light Chain Levels than Patients with Schizophrenia. Asian J. Psychiatr..

[68] Rothberg R.L., Azhari N., Haug N.A., Dakwar E. (2021). Mystical-Type Experiences Occasioned by Ketamine Mediate Its Impact on at-Risk Drinking: Results from a Randomized, Controlled Trial. J. Psychopharmacol..

[69] Husain M.I., Ledwos N., Fellows E., Baer J., Rosenblat J.D., Blumberger D.M., Mulsant B.H., Castle D.J. (2023). Serotonergic Psychedelics for Depression: What Do We Know about Neurobiological Mechanisms of Action?. Front. Psychiatry.

[70] Vollenweider F.X., Preller K.H. (2020). Psychedelic Drugs: Neurobiology and Potential for Treatment of Psychiatric Disorders. Nat. Rev. Neurosci..

[71] Xiang C., Hong S.M., Zhao B., Pi H., Du F., Lu X., Tang Y., Shen N., Yang C., Wang R. (2024). Fibroblast Expression of Neurotransmitter Receptor HTR2A Associates with Inflammation in Rheumatoid Arthritis Joint. Clin. Exp. Med..

[72] Leó N-Ponte M., Ahern G.P., O’connell P.J. (2007). Serotonin Provides an Accessory Signal to Enhance T-Cell Activation by Signaling through the 5-HT 7 Receptor. Blood.

[73] Idova G.V., Alperina E.L., Cheido M.A. (2012). Contribution of Brain Dopamine, Serotonin and Opioid Receptors in the Mechanisms of Neuroimmunomodulation: Evidence from Pharmacological Analysis. Int. Immunopharmacol..

[74] do Nascimento Sousa D., de Azevedo M., Santos M.L., Borges T.K.D.S., de Oliveira D.M., Caldas E.D. (2025). Immunomodulatory and Behavioral Effects of Ayahuasca and N, N-Dimethyltryptamine in a Rat Model of Lipopolysaccharide-Induced Depression. Metab. Brain Dis..

[75] Floris G., Dabrowski K.R., Zanda M.T., Daws S.E. (2025). Psilocybin Reduces Heroin Seeking Behavior and Modulates Inflammatory Gene Expression in the Nucleus Accumbens and Prefrontal Cortex of Male Rats. Mol. Psychiatry.

[76] Burmester D.R., Madsen M.K., Szabo A., Aripaka S.S., Stenbæk D.S., Frokjaer V.G., Elfving B., Mikkelsen J.D., Knudsen G.M., Fisher P.M.D. (2023). Subacute Effects of a Single Dose of Psilocybin on Biomarkers of Inflammation in Healthy Humans: An Open-Label Preliminary Investigation. Compr. Psychoneuroendocrinol..

[77] Idell R.D., Florova G., Komissarov A.A., Shetty S., Girard R.B.S., Idell S. (2017). The Fibrinolytic System: A New Target for Treatment of Depression with Psychedelics. Med. Hypotheses.

[78] Graça S.C., Bustelli I.B., dos Santos É.V., Fernandes C.G., Lanaro R., Stilhano R.S., Linardi A., Caetano A.L. (2025). Banisteriopsis Caapi Extract: Implications for Neuroinflammatory Pathways in Locus Coeruleus Lesion Rodent Model. J. Ethnopharmacol..

[79] Rudin D., Areesanan A., Liechti M.E., Gründemann C. (2023). Classic Psychedelics Do Not Affect T Cell and Monocyte Immune Responses. Front. Psychiatry.

[80] Zhang J., Ma L., Wan X., Shan J., Qu Y., Hashimoto K. (2021). (R)-Ketamine Attenuates LPS-Induced Endotoxin-Derived Delirium through Inhibition of Neuroinflammation. Psychopharmacology.

[81] Nau F., Miller J., Saravia J., Ahlert T., Yu B., Happel K.I., Cormier S.A., Nichols C.D. (2015). Serotonin 5-HT 2 Receptor Activation Prevents Allergic Asthma in a Mouse Model. Am. J. Physiol. Lung Cell Mol. Physiol..

[82] Smith J.A., Das A., Ray S.K., Banik N.L. (2012). Role of Pro-Inflammatory Cytokines Released from Microglia in Neurodegenerative Diseases. Brain Res. Bull..

[83] Gruchot J., Weyers V., Göttle P., Förster M., Kremer D., Hartung H.P., Küry P. (2019). The Molecular Basis for Remyelination Failure in Multiple Sclerosis. Cells.

[84] Absinta M., Maric D., Gharagozloo M., Garton T., Smith M.D., Jin J., Fitzgerald K.C., Song A., Liu P., Lin J.P. (2021). A Lymphocyte–Microglia–Astrocyte Axis in Chronic Active Multiple Sclerosis. Nature.

[85] Laabi S., LeMmon C., Vogel C., Chacon M., Jimenez V.M. (2025). Psilocybin and Psilocin Regulate Microglial Immunomodulation and Support Neuroplasticity via Serotonergic and AhR Signaling. Int. Immunopharmacol..

[86] Low Z.X.B., Ng W.S., Lim E.S.Y., Goh B.H., Kumari Y. (2024). The Immunomodulatory Effects of Classical Psychedelics: A Systematic Review of Preclinical Studies. Prog. Neuropsychopharmacol. Biol. Psychiatry.

[87] Ponath G., Park C., Pitt D. (2018). The Role of Astrocytes in Multiple Sclerosis. Front. Immunol..

[88] Casili G., Lanza M., Filippone A., Cucinotta L., Paterniti I., Repici A., Capra A.P., Cuzzocrea S., Esposito E., Campolo M. (2022). Dimethyl Fumarate (DMF) Alleviated Post-Operative (PO) Pain through the N-Methyl-d-Aspartate (NMDA) Receptors. Antioxidants.

[89] Ly C., Greb A.C., Cameron L.P., Wong J.M., Barragan E.V., Wilson P.C., Burbach K.F., Soltanzadeh Zarandi S., Sood A., Paddy M.R. (2018). Psychedelics Promote Structural and Functional Neural Plasticity. Cell Rep..

[90] Faissner S., Plemel J.R., Gold R., Yong V.W. (2019). Progressive Multiple Sclerosis: From Pathophysiology to Therapeutic Strategies. Nat. Rev. Drug Discov..

[91] Erkizia-Santamaría I., Horrillo I., Martínez-Álvarez N., Pérez-Martínez D., Rivero G., Erdozain A.M., Meana J.J., Ortega J.E. (2025). Evaluation of Behavioural and Neurochemical Effects of Psilocybin in Mice Subjected to Chronic Unpredictable Mild Stress. Transl. Psychiatry.

[92] Brunello C.A., Cannarozzo C., Castrén E. (2024). Rethinking the Role of TRKB in the Action of Antidepressants and Psychedelics. Trends Neurosci..

[93] Ben-Tal T., Pogodin I., Botvinnik A., Lifschytz T., Heresco-Levy U., Lerer B. (2025). Synergistic Behavioral and Neuroplastic Effects of Psilocybin-NMDAR Modulator Administration. Transl. Psychiatry.

[94] Ornelas I.M., Cini F.A., Wießner I., Marcos E., Araújo D.B., Goto-Silva L., Nascimento J., Silva S.R.B., Costa M.N., Falchi M. (2022). Nootropic Effects of LSD: Behavioral, Molecular and Computational Evidence. Exp. Neurol..

[95] Costa M.N., Goto-Silva L., Nascimento J.M., Domith I., Karmirian K., Feilding A., Trindade P., Martins-De-Souza D., Rehen S.K. (2024). LSD Modulates Proteins Involved in Cell Proteostasis, Energy Metabolism and Neuroplasticity in Human Cerebral Organoids. ACS Omega.

[96] Lipton J.O., Sahin M. (2014). The Neurology of MTOR. Neuron.

[97] Martín-Guerrero S.M., Alonso P., Iglesias A., Cimadevila M., Brea J., Loza M.I., Casado P., Martín-Oliva D., Cutillas P.R., González-Maeso J. (2021). His452Tyr Polymorphism in the Human 5-HT2A Receptor Affects Clozapine-Induced Signaling Networks Revealed by Quantitative Phosphoproteomics. Biochem. Pharmacol..

[98] Gharagozloo M., Bannon R., Calabresi P.A. (2022). Breaking the Barriers to Remyelination in Multiple Sclerosis. Curr. Opin. Pharmacol..

[99] Gautier H.O.B., Evans K.A., Volbracht K., James R., Sitnikov S., Lundgaard I., James F., Lao-Peregrin C., Reynolds R., Franklin R.J.M. (2015). Neuronal Activity Regulates Remyelination via Glutamate Signalling to Oligodendrocyte Progenitors. Nat. Commun..

[100] Funk D., Araujo J., Slassi M., Lanthier J., Atkinson J., Feng D., Lau W., Lê A., Higgins G.A. (2024). Effect of a Single Psilocybin Treatment on Fos Protein Expression in Male Rat Brain. Neuroscience.

[101] EI Waly B., Macchi M., Cayre M., Durbec P. (2014). Oligodendrogenesis in the Normal and Pathological Central Nervous System. Front. Neurosci..

[102] Irvine K.A., Blakemore W.F. (2008). Remyelination Protects Axons from Demyelination-Associated Axon Degeneration. Brain.

[103] Grieco S.F., Castrén E., Knudsen G.M., Kwan A.C., Olson D.E., Zuo Y., Holmes T.C., Xu X. (2022). Psychedelics and Neural Plasticity: Therapeutic Implications. J. Neurosci. Soc. Neurosci..

[104] Maas D.A., Angulo M.C. (2021). Can Enhancing Neuronal Activity Improve Myelin Repair in Multiple Sclerosis?. Front. Cell Neurosci..

[105] Johnson M.W., Griffiths R.R., Hendricks P.S., Henningfield J.E. (2018). The Abuse Potential of Medical Psilocybin According to the 8 Factors of the Controlled Substances Act. Neuropharmacology.

[106] Romeo B., Kervadec E., Fauvel B., Strika-Bruneau L., Amirouche A., Verroust V., Piolino P., Benyamina A. (2024). Safety and Risk Assessment of Psychedelic Psychotherapy: A Meta-Analysis and Systematic Review. Psychiatry Res..

[107] Bremler R., Katati N., Shergill P., Erritzoe D., Carhart-Harris R.L. (2023). Case Analysis of Long-Term Negative Psychological Responses to Psychedelics. Sci. Rep..

[108] Marrie R.A., Fisk J., Tremlett H., Wolfson C., Warren S., Blanchard J., Patten S.B. (2016). Neurology^®^ Clinical Practice Differing Trends in the Incidence of Vascular Comorbidity in MS and the General Population. Neurol. Clin. Pract..

[109] Nahlawi A., Ptaszek L.M., Ruskin J.N. (2025). Cardiovascular Effects and Safety of Classic Psychedelics. Nat. Cardiovasc. Res..

[110] Rouaud A., Calder A.E., Hasler G. (2024). Microdosing Psychedelics and the Risk of Cardiac Fibrosis and Valvulopathy: Comparison to Known Cardiotoxins. J. Psychopharmacol..

[111] Tagen M., Mantuani D., van Heerden L., Holstein A., Klumpers L.E., Knowles R. (2023). The Risk of Chronic Psychedelic and MDMA Microdosing for Valvular Heart Disease. J. Psychopharmacol..

[112] Downey A.E., Chaphekar A.V., Woolley J., Raymond-Flesch M. (2024). Psilocybin Therapy and Anorexia Nervosa: A Narrative Review of Safety Considerations for Researchers and Clinicians. J. Eat. Disord..

[113] Jellinger K.A. (2024). Depression and Anxiety in Multiple Sclerosis. Review of a Fatal Combination. J. Neural Transm..

[114] Mustač F., Pašić H., Medić F., Bjedov B., Vujević L., Alfirević M., Vidrih B., Tudor K.I., Bošnjak Pašić M. (2021). Anxiety and Depression as Comorbidities of Multiple Sclerosis. Psychiatr. Danub..

[115] Marrocu A., Kettner H., Weiss B., Zeifman R.J., Erritzoe D., Carhart-Harris R.L. (2024). Psychiatric Risks for Worsened Mental Health after Psychedelic Use. J. Psychopharmacol..

[116] Simonsson O., Goldberg S.B., Chambers R., Osika W., Simonsson C., Hendricks P.S. (2023). Psychedelic Use and Psychiatric Risks. Psychopharmacology.

[117] Elfrink S., Bergin L. (2025). Psychedelic Iatrogenic Structural Dissociation: An Exploratory Hypothesis on Dissociative Risks in Psychedelic Use. Front. Psychol..

[118] Borkel L.F., Rojas-Hernández J., Quintana-Hernández D.J., Henríquez-Hernández L.A. (2025). Therapeutic Benefit versus Epistemic Risk: Need for Empirical Research in Psychedelic Epistemology. J. Psychiatr. Res..

[119] Caporuscio C., Fink S.B. (2024). Epistemic Risk Reduction in Psychedelic-Assisted Therapy.

[120] Pacilio R.M., Geller J.A. (2025). Persistent Psychosis Associated with Intravenous Ketamine in a Patient Using Cannabis: A Case Report and Literature Review. J. Clin. Psychopharmacol..

[121] Bavato F., Quednow B.B. (2025). Ketamine Addiction in Europe: Any Risks on the Horizons?. Eur. Neuropsychopharmacol..

[122] Morton E., Sakai K., Ashtari A., Pleet M., Michalak E.E., Woolley J. (2023). Risks and Benefits of Psilocybin Use in People with Bipolar Disorder: An International Web-Based Survey on Experiences of ‘Magic Mushroom’ Consumption. J. Psychopharmacol..

[123] Pérez L.P., González R.S., Lázaro E.B. (2015). Treatment of Mood Disorders in Multiple Sclerosis. Curr. Treat. Options Neurol..

[124] Soto-Angona Ó., Fortea A., Fortea L., Martínez-Ramírez M., Santamarina E., López F.J.G., Knudsen G.M., Ona G. (2024). Do Classic Psychedelics Increase the Risk of Seizures? A Scoping Review. Eur. Neuropsychopharmacol..

[125] Perkins D., Sarris J., Rossell S., Bonomo Y., Forbes D., Davey C., Hoyer D., Loo C., Murray G., Hood S. (2021). Medicinal Psychedelics for Mental Health and Addiction: Advancing Research of an Emerging Paradigm. Aust. N. Z. J. Psychiatry.

[126] Jones N.T., Zahid Z., Grady S.M., Sultan Z.W., Zheng Z., Razidlo J., Banks M.I., Wenthur C.J. (2023). Transient Elevation of Plasma Glucocorticoids Supports Psilocybin-Induced Anxiolysis in Mice. ACS Pharmacol. Transl. Sci..

[127] Rucker J.J.H., Iliff J., Nutt D.J. (2018). Psychiatry & the Psychedelic Drugs. Past, Present & Future. Neuropharmacology.

[128] Schonholz S.M., Appel J.M., Bursztajn H.J., Nair M., MacIntyre M.R. (2024). Legal and Ethics Concerns of Psilocybin as Medicine. J. Am. Acad. Psychiatry Law.

[129] Siegel J.S., Daily J.E., Perry D.A., Nicol G.E. (2023). Psychedelic Drug Legislative Reform and Legalization in the US. JAMA Psychiatry.

[130] Barnett B.S., Anand A., Dewey E.N., Smith D., Nayak S.M., Bruckman D., Weleff J. (2024). Perceived Risk of Trying Lysergic Acid Diethylamide in the United States from 2015 to 2019: Are Americans Assessing Lysergic Acid Diethylamide’s Risk Profile More Favorably?. Psychedelic Med..

[131] Xenakis S.N., Shannon S.M. (2024). What Is Needed for the Roll-out of Psychedelic Treatments?. Curr. Opin. Psychiatry.

[132] Sheppard B. (2021). A Trip Through Employment Law: Protecting Therapeutic Psilocybin Users in the Workplace. J. Law. Health.

[133] Aday J.S., Barnett B.S., Grossman D., Murnane K.S., Nichols C.D., Hendricks P.S. (2023). Psychedelic Commercialization: A Wide-Spanning Overview of the Emerging Psychedelic Industry. Psychedelic Med..

[134] Li S., Kurtzweil T., Shams S., Pratt A., Rudi S. (2024). Intellectual Property of Psychedelics for Addiction Treatment: Enabling Access and Protecting Innovation Opportunities Through Preserving the Public Domain. J. Stud. Alcohol. Drugs.

[135] Belouin S.J., Averill L.A., Henningfield J.E., Xenakis S.N., Donato I., Grob C.S., Berger A., Magar V., Danforth A.L., Anderson B.T. (2022). Policy Considerations That Support Equitable Access to Responsible, Accountable, Safe, and Ethical Uses of Psychedelic Medicines. Neuropharmacology.

[136] Oates E. (1988). Spectrum of Appearance of Hyperostosis Frontalis Interna on In-111 Leukocyte Scans. Clin. Nucl. Med..

[137] Wang E., Mathai D.S., Gukasyan N., Nayak S., Garcia-Romeu A. (2024). Knowledge, Attitudes, and Concerns about Psilocybin and MDMA as Novel Therapies among U.S. Healthcare Professionals. Sci. Rep..

[138] Pierre M.S., Standing L., Herman Y., Haden M., Walsh Z. (2024). Patients’ Experiences Discussing Psychedelics for Therapeutic Purposes with Physicians and Other Health Care Providers. Psychedelic Med..

[139] Boehnke K.F., Cox K., Weston C., Herberholz M., Glynos N., Kolbman N., Fields C.W., Barron J., Kruger D.J. (2023). Slouching towards Engagement: Interactions between People Using Psychedelics Naturalistically and Their Healthcare Providers. Front. Psychiatry.

[140] Celidwen Y., Redvers N., Githaiga C., Calambás J., Añaños K., Evanjuanoy Chindoy M., Vitale R., Nelson Rojas J., Mondragón D., Vázquez Rosalío Y. (2022). Ethical Principles of Traditional Indigenous Medicine to Guide Western Psychedelic Research and Practice. Lancet Reg. Health Am..

[141] Spriggs M.J., Murphy-Beiner A., Murphy R., Bornemann J., Thurgur H., Schlag A.K. (2023). ARC: A Framework for Access, Reciprocity and Conduct in Psychedelic Therapies. Front. Psychol..

[142] Back A.L., Freeman-Young T.K., Morgan L., Sethi T., Baker K.K., Myers S., McGregor B.A., Harvey K., Tai M., Kollefrath A. (2024). Psilocybin Therapy for Clinicians with Symptoms of Depression from Frontline Care During the COVID-19 Pandemic: A Randomized Clinical Trial. JAMA Netw. Open.

[143] Cavarra M., Falzone A., Ramaekers J.G., Kuypers K.P.C., Mento C. (2022). Psychedelic-Assisted Psychotherapy—A Systematic Review of Associated Psychological Interventions. Front. Psychol..

[144] Mintz K.T., Gammer B., Khan A.J., Shaub G., Levine S., Sisti D. (2022). Physical Disability and Psychedelic Therapies: An Agenda for Inclusive Research and Practice. Front. Psychiatry.

[145] Villiger D. (2024). Personal Psychedelic Experience of Psychedelic Therapists During Training: Should It Be Required, Optional, or Prohibited?. Int. Rev. Psychiatry.

[146] Harrison T.R., Faber S.C., Zare M., Fontaine M., Williams M.T. (2025). Wolves Among Sheep: Sexual Violations in Psychedelic-Assisted Therapy. Am. J. Bioeth..

[147] Bender D.A., Nayak S.M., Siegel J.S., Hellerstein D.J., Ercal B.C., Lenze E.J. (2025). The Role of Touch in Psychedelic Therapy: Perspectives from a Survey of Practitioners in Research Settings. Am. J. Psychother..

[148] Buchman D., Rosenbaum D. (2024). Psychedelics in PERIL: The Commercial Determinants of Health, Financial Entanglements and Population Health Ethics. Public Health Ethics.

[149] Olson D.E. (2022). Biochemical Mechanisms Underlying Psychedelic-Induced Neuroplasticity. Biochemistry.

[150] Sellers E.M., Romach M.K. (2023). Psychedelics: Science Sabotaged by Social Media. Neuropharmacology.

[151] Bellman V. (2024). Review of Psilocybin Use for Depression among Cancer Patients After Approval in Oregon. Cancers.

[152] Korthuis P.T., Hoffman K., Wilson-Poe A.R., Luoma J.B., Bazinet A., Pertl K., Morgan D.L., Cook R.R., Bielavitz S., Myers R. (2024). Developing the Open Psychedelic Evaluation Nexus Consensus Measures for Assessment of Supervised Psilocybin Services: An e-Delphi Study. J. Psychopharmacol..

[153] Flanagan T.W., Nichols C.D., Barrett F.S., Preller K.H. (2022). Psychedelics and Anti-Inflammatory Activity in Animal Models. Disruptive Psychopharmacology.

[154] Polito V., Stevenson R.J. (2019). A Systematic Study of Microdosing Psychedelics. PLoS ONE.

[155] Anderson T., Petranker R., Christopher A., Rosenbaum D., Weissman C., Dinh-Williams L.A., Hui K., Hapke E. (2019). Psychedelic Microdosing Benefits and Challenges: An Empirical Codebook. Harm Reduct. J..

[156] Bavato F., Stamatakos S., Yde Ohki C.M., Seifritz E., Romualdi P., Grünblatt E., Quednow B.B. (2022). Brain-Derived Neurotrophic Factor Protects Serotonergic Neurons against 3,4-Methylenedioxymethamphetamine (“Ecstasy”) Induced Cytoskeletal Damage. J. Neural Transm..

[157] Franklin R.J.M., Ffrench-Constant C. (2008). Remyelination in the CNS: From Biology to Therapy. Nat. Rev. Neurosci..

[158] Rudick R.A., Larocca N., Hudson L.D. (2013). Multiple Sclerosis Outcome Assessments Consortium: Genesis and Initial Project Plan. Mult. Scler. J..

[159] Muraro P.A., Mariottini A., Greco R., Burman J., Iacobaeus E., Inglese M., Snowden J.A., Alexander T., Amato M.P., Bø L. (2025). Autologous Haematopoietic Stem Cell Transplantation for Treatment of Multiple Sclerosis and Neuromyelitis Optica Spectrum Disorder—Recommendations from ECTRIMS and the EBMT. Nat. Rev. Neurol..

[160] Dhib-Jalbut S., Marks S. (2010). Interferon-β Mechanisms of Action in Multiple Sclerosis. Neurology.

[161] Aharoni R. (2013). The Mechanism of Action of Glatiramer Acetate in Multiple Sclerosis and Beyond. Autoimmun. Rev..

[162] Banks W.A., Rhea E.M., Reed M.J., Erickson M.A. (2024). The Penetration of Therapeutics across the Blood-Brain Barrier: Classic Case Studies and Clinical Implications. Cell Rep. Med..

[163] Xiong B., Wang Y., Chen Y., Xing S., Liao Q., Chen Y., Li Q., Li W., Sun H. (2021). Strategies for Structural Modification of Small Molecules to Improve Blood-Brain Barrier Penetration: A Recent Perspective. J. Med. Chem..

[164] Evens R., Schmidt M.E., Majić T., Schmidt T.T. (2023). The Psychedelic Afterglow Phenomenon: A Systematic Review of Subacute Effects of Classic Serotonergic Psychedelics. Ther. Adv. Psychopharmacol..

[165] Henner R.L., Keshavan M.S., Hill K.P. (2022). Review of Potential Psychedelic Treatments for PTSD. J. Neurol. Sci..

[166] Valdez T., Patel V., Senesombath N., Hatahet-Donovan Z., Hornick M. (2024). Therapeutic Potential of Psychedelic Compounds for Substance Use Disorders. Pharmaceuticals.

[167] Wallach J., Cao A.B., Calkins M.M., Heim A.J., Lanham J.K., Bonniwell E.M., Hennessey J.J., Bock H.A., Anderson E.I., Sherwood A.M. (2023). Identification of 5-HT2A Receptor Signaling Pathways Associated with Psychedelic Potential. Nat. Commun..

[168] Zhou L., Kodidela S., Godse S., Thomas-Gooch S., Kumar A., Raji B., Zhi K., Kochat H., Kumar S. (2022). Targeted Drug Delivery to the Central Nervous System Using Extracellular Vesicles. Pharmaceuticals.

[169] Lu J., Tjia M., Mullen B., Cao B., Lukasiewicz K., Shah-Morales S., Weiser S., Cameron L.P., Olson D.E., Chen L. (2021). An Analog of Psychedelics Restores Functional Neural Circuits Disrupted by Unpredictable Stress. Mol. Psychiatry.

[170] Caspani G., Ruffell S.G.D., Tsang W.F., Netzband N., Rohani-Shukla C., Swann J.R., Jefferies W.A. (2024). Mind over Matter: The Microbial Mindscapes of Psychedelics and the Gut-Brain Axis. Pharmacol. Res..

[171] Szabo A., Frecska E. (2016). Dimethyltryptamine (DMT): A Biochemical Swiss Army Knife in Neuroinflammation and Neuroprotection?. Neural Regen. Res..

